# Transcription Factor Blimp-1: A Central Regulator of Oxidative Stress and Metabolic Reprogramming in Chronic Inflammatory Diseases

**DOI:** 10.3390/antiox14020183

**Published:** 2025-02-04

**Authors:** Aline Yen Ling Wang, Ana Elena Aviña, Yen-Yu Liu, Yun-Ching Chang, Huang-Kai Kao

**Affiliations:** 1 Center for Vascularized Composite Allotransplantation, Chang Gung Memorial Hospital, Taoyuan 333, Taiwan; aviestlani@hotmail.com (A.E.A.); louis881128@gmail.com (Y.-Y.L.); 2 International PhD Program in Medicine, College of Medicine, Taipei Medical University, Taipei 110, Taiwan; 3 Department of Health Industry Technology Management, Chung Shan Medical University, Taichung 402, Taiwan; changyc@csmu.edu.tw; 4 Department of Medical Research, Chung Shan Medical University Hospital, Taichung 402, Taiwan; 5 Department of Plastic and Reconstructive Surgery, Chang Gung Memorial Hospital, Taoyuan 333, Taiwan; kai3488@gmail.com; 6 College of Medicine, Chang Gung University, Taoyuan 333, Taiwan

**Keywords:** Blimp-1, IL-10, reactive oxygen species, oxidative stress, antioxidant pathways, chronic inflammatory diseases, metabolic reprogramming

## Abstract

B-lymphocyte-induced maturation protein 1 (Blimp-1) is a transcription factor that, among other functions, modulates metabolism and helps to regulate antioxidant pathways, which is important in the context of chronic inflammatory diseases like diabetes, cardiovascular disease, and autoimmune disease. In immune cell function, Blimp-1 has a modulatory role in the orchestration of metabolic reprogramming and as a promoter of anti-inflammatory cytokines, including IL-10, responsible for modulating oxidative stress and immune homeostasis. Moreover, Blimp-1 also modulates key metabolic aspects, such as glycolysis and fatty acid oxidation, which regulate reactive oxygen species levels, as well as tissue protection. This review depicts Blimp-1 as an important regulator of antioxidant defenses and anti-inflammation and suggests that the protein could serve as a therapeutic target in chronic inflammatory and metabolic dysregulation conditions. The modulation of Blimp-1 in diseases such as diabetic coronary heart disease and atherosclerosis could alleviate oxidative stress, augment the protection of tissues, and improve disease outcomes. The therapeutic potential for the development of new treatments for these chronic conditions lies in the synergy between the regulation of Blimp-1 and antioxidant therapies, which are future directions that may be pursued. This review emphasizes Blimp-1’s emerging importance as a novel regulator in the pathogenesis of inflammatory diseases, providing new opportunities for therapeutic intervention.

## 1. Introduction

B-lymphocyte-induced maturation protein 1 (Blimp-1) is encoded by the *Prdm1* gene, a zinc finger containing a transcriptional repressor that controls gene expression by repressing important transcriptional programs. It has emerged as a critical regulator of immune function and metabolism [1]. Blimp-1 was first identified for its role in driving the differentiation of plasma cells in B lymphocytes and has since been implicated in a diverse range of immune cell types: B lymphocytes, T lymphocytes, dendritic cells (DCs), and innate lymphoid cells (ILCs) [1,2]. Cellular processes as varied as immune regulation and metabolic control are orchestrated by it in the maintenance of tissue homeostasis and immune tolerance. Blimp-1 modulates the expression of key cytokines in the context of immune regulation, including interleukin-10 (IL-10), particularly in regulatory T cells (Tregs) and other immune cell subsets. The human *PRDM1* gene and the mouse *Prdm1* gene both encode transcriptional repressors that are of crucial importance in immune regulation and metabolism; Blimp-1, after which these genes were named, is the protein product. Highly conserved between species, but each with slight distinctions in their gene structure and protein domains, their study offers insight into the species-specific roles of Blimp-1 [3,4]. Herein, we summarize the features common to human and mouse Blimp-1, as well as their differences (Table 1). In multiple chronic diseases, IL-10 is key to controlling inflammation, preventing autoimmunity, and promoting tissue repair [1,5]. Moreover, Blimp-1 also suppresses pro-inflammatory T helper cell type differentiation, including T helper (Th)1 [6,7], Th17, and Th9 cells, and is essential in maintaining immune homeostasis [8,9,10].

Excessive reactive oxygen species (ROS), byproducts of normal metabolic processes, lead to oxidative stress and contribute to tissue damage and inflammation. Elevated ROS levels are an underlying characteristic of chronic inflammatory diseases such as rheumatoid arthritis (RA), inflammatory bowel disease (IBD), and diabetes and may actually contribute to worsening the disease [11,12,13,14,15,16,17]. In these diseases, the body’s antioxidant defenses, such as enzymes including superoxide dismutase (SOD) and heme oxygenase-1 (HO-1), are critical in minimizing oxidative damage [12,13,18,19]. Antioxidant responses regulated by Blimp-1 itself or signaling molecules such as IL-10 are implicated. For illustration, studies have shown that the overexpression of Blimp-1 can diminish some markers of oxidative stress in models of diabetic coronary heart disease (DM-CHD), including, for example, malondialdehyde (MDA) levels or increased antioxidant SOD activity, possibly promoting protection against oxidant damage [10].

It is becoming clear that immune regulation, metabolism, and oxidative stress are connected. Blimp-1 is one of a group of transcription factors that may act as integrators of immune signals and metabolic pathways and modulate cellular responses to stress and inflammation. Blimp-1’s role in coordinating immune responses is closely linked with its regulatory function in metabolism. For example, in the adipose tissue, Tregs driving the Blimp-1-mediated production of IL-10 negatively regulate metabolic pathways that drive obesity and insulin resistance [5]. In addition, Blimp-1 reduces energy-expending activities, such as adipocyte browning, which leads to increased energy expenditure and thermogenesis. Blimp-1 functions critically in DCs to regulate the expression of genes that determine antigen presentation and cytokine production [1]. Blimp-1’s expression in DCs has also recently been shown to promote the expression of amphiregulin (AREG), a growth factor that is critically involved in tissue repair and homeostasis, especially in conditions of oxidative stress and inflammation [20]. In addition, metabolic processes in immune cells themselves, e.g., the metabolic reprogramming of group 2 innate lymphoid cells (ILC2s), by shifting from fatty acid oxidation to glycolysis in response to inflammation, are under the regulatory control of Blimp-1 [21].

Recently, Blimp-1 has been identified as a novel regulator of antioxidant pathways, which it achieves through multiple avenues. The induction of IL-10, a potent anti-inflammatory and cytoprotective substance, is involved in one of the key pathways [1,2]. The expression of antioxidant enzymes, such as SOD, is promoted by IL-10, which neutralizes ROS and avoids cellular damage [22,23,24]. Of specific relevance to chronic diseases driven by oxidative stress such as atherosclerosis [10], Blimp-1 modulates cellular antioxidant responses. In DM-CHD models, Blimp-1 decreases markers of oxidative stress and atherosclerotic plaque formation, thus offering a therapeutic target for diseases associated with chronic inflammation and oxidative damage [10]. Blimp-1 is a central regulator at the crossroads of immunity, metabolism, and oxidative stress. Because of its capacity to modulate immune responses, cellular metabolism, and antioxidant pathways, it may be a novel therapeutic target in chronic inflammatory diseases. Blimp-1 enhances IL-10 production, leading to decreased pathogenic inflammation while also influencing key antioxidant enzymes that reduce oxidative damage, providing a pathway toward restoring oxidative homeostasis in chronic inflammation. With an increasing number of its functions being uncovered, Blimp-1 could become a critical modulator of immune and metabolic health that informs the treatment of chronic diseases.

Recently, considerable progress has been made in elucidating the function of Blimp-1 in individual contexts, but an integrated role for Blimp-1 in regulating oxidative stress, metabolism, and the immune system as a whole has yet to be described. A wide range of inflammatory diseases, ranging from RA to atherosclerosis to diabetes, are driven by chronic oxidative stress. The transcription factor Blimp-1 has recently come to the fore as a key regulator at the nexus between immune activity, metabolism, and oxidative stress. Although single studies have investigated Blimp-1’s function in immune regulation, IL-10 production, and metabolic control, an overall view of the way in which Blimp-1 integrates all of these functions into a single network to regulate oxidative stress and inflammation remains unexplored. Second, synthesizing this evidence fills a critical gap in explaining the ability of Blimp-1: (1) Blimp-1 drives IL-10 production to couple immune regulation with antioxidant defense, including the modulation of SOD and HO-1; (2) Blimp-1 drives the metabolic reprogramming of glycolysis and fatty acid oxidation in immune cells, influencing the production of ROS and tissue homeostasis; (3) these may act as a potential therapeutic target for diseases caused by chronic oxidative stress and inflammation. Blimp-1 appears to be the central integrator of the body’s response to oxidative stress because of its unique ability to regulate pathways involved in both immune and metabolic functions. This review highlights how these unique functions of Blimp-1 may be utilized to generate alternative therapeutic strategies targeting oxidative stress and chronic inflammation and thereby potentially aid the management of such disorders.

## 2. Blimp-1 as a Regulatory Transcription Factor

### 2.1. Regulation in B Cells

Blimp-1 is the master regulator of B cells, regulating the transition of activated B cells to plasma cells, which is indispensable for antibody production [25,26,27,28]. It achieves this by repressing critical transcription factors responsible for maintaining B cell identity, such as Pax5 and Bcl-6, and upregulating genes that empower plasma cell function [4,28,29,30]. However, among these, Xbp-1 has a crucial role in the production of the secretory apparatus required for antibody secretion [27,31]. Experimental Blimp-1 knockout models also illustrate the critical importance of Blimp-1 in B cells, since B cells lacking Blimp-1 do not differentiate into plasma cells and consequently have severely compromised humoral immune responses [31,32,33]. Blimp-1 knockout studies clearly show that Blimp-1 is required for B-cell-mediated immune responses, since the absence of Blimp-1 results in the failure to create long-lived plasma cells and therefore to mount appropriate antibody responses [27]. Furthermore, overexpression studies have proven that Blimp-1 is a master regulator of plasma cell differentiation [27,28,34,35]. The artificial overexpression of Blimp-1 in mature B cells drives their differentiation to plasma cells, even in the absence of normal differentiation signals. This adds to the status of Blimp-1 as a central regulator of B cell fate [31].

The regulation of B cell differentiation by Blimp-1 is additionally dependent on its reciprocal relation with Bcl-6, another key transcription factor for germinal center B cell formation [28]. Bcl-6 supports the generation and maintenance of germinal centers for the suitable B cell class-switch recombination to raise antibody specificity [28]. In this antagonistic dynamic, Bcl-6 directly suppresses Blimp-1 in order to maintain germinal center reactions by allowing B cells to proliferate, undergo somatic hypermutation, and select better-affinity antibodies [28]. Meanwhile, Blimp-1 blocks Bcl-6 activity to arrest germinal center reactions and productively drive B cell differentiation to plasma cells, which no longer undergo further proliferation but secrete massive amounts of antibodies [27]. Blimp-1 inhibits c-Myc, Pax5, and Bcl-6 and prevents cell proliferation until plasma cells fully differentiate into the non-dividing state with high rates of antibody production [4]. In the absence of this suppression, B cells could not exit the germinal center, and the long-lived plasma cells necessary for sustained immune protection would not develop. Finally, Blimp-1 is the main transcription factor of the terminal differentiation of B cells into plasma cells [31]. Blimp-1’s interaction with Bcl-6 and regulation of key gene programs ensure that B cell germinal center cells without a function in antibody production become fully functional, antibody-producing plasma cells. Blimp-1 is essential for both acute immune responses and the generation of long-term immunity, and it is necessary to maintain the careful balance between the activation of these pathways and immune homeostasis in B cells.

### 2.2. Regulation in T Cells

Blimp-1 is an important and versatile regulator of effector and memory cell differentiation in T cells [2,36,37]. Both CD4^+^ and CD8^+^ T cells express Blimp-1, which directs the transcriptional programs that are important for proper T cell function [28,38,39]. As such, its function in regulating cytokine expression plays a central role in directing the development of different T cell subsets. The regulation of the expression of cytokines such as IL-2, IL-10, and IFN-γ by Blimp-1 is important to fine tune immune responses and effector functions across T cell lineages [33]. Blimp-1 is required in Tregs to support the expression of IL-10 [2], an anti-inflammatory cytokine [40,41] that is critical for immune homeostasis. IL-10 is critical in the regulation of inflammation and the prevention of immune-mediated tissue damage. Indeed, Blimp-1 drives the IL-10 production necessary for Tregs’ suppression of excessive immune responses and blocks the pathogenic Th17 activity [28,42]. Blimp-1 prevents autoimmune disorders by preventing the overactivation of Th17 cells and the breakdown of immune tolerance. Blimp-1 knockout T cell models indicate a promotion effect to develop spontaneous inflammatory diseases [4], demonstrating that Blimp-1 is critical in disabling immune hyperactivation and regulating cell differentiation.

In CD8^+^ T cells, Blimp-1 is critical for the production of both short-lived effector cells and memory precursors [2]. Blimp-1 facilitates the differentiation of effector CD8^+^ T cells, which form a critical arm of the immediate cytotoxic response to viral and cancerous challenges. Blimp-1 knockout studies show that CD8^+^ T cells harboring no Blimp-1 produce fewer cytokines, migrate poorly, and have a reduced capacity for cytotoxicity, which impairs their ability to clear infections and tumors [33]. For example, CD8^+^ T cells from Blimp-1-deficient mice fail to mount proper immune defense against viral infections due to the compromised ability of Blimp-1-deficient CD8^+^ T cells to maintain cytotoxicity. The generation of long-lived memory T cells is dependent on Blimp-1 in memory CD8^+^ T cells [39,43,44]. These cells are important for a rapid response when humans are re-exposed to pathogens. Knockout models of Blimp-1 indicate that Blimp-1 maintains functional memory CD8^+^ T cells at reduced numbers [2], thereby impairing long-term immune surveillance and protection from reinfections and tumorigenesis. The lack of sustained memory precursors in antigen stimulation, despite the failure of Blimp-1-deficient CD8^+^ T cells to establish such precursors, demonstrates the importance of Blimp-1 in functional T cells in multiple immune contexts. In sum, Blimp-1 is indispensable for T cell immunity, integrating the responses of Tregs, CD4^+^, and CD8^+^ T cells. Its regulation of cytokine expression, downregulation of immune hyperactivation, and development of effector and memory T cells are essential for immune tolerance, as well as for the induction of effective immune responses against infections and malignancies.

### 2.3. Regulation in Mononuclear Phagocyte System

In addition to its previously described function in adaptive immunity, Blimp-1’s regulation extends beyond this to innate immune cells such as DCs and macrophages [1]. Blimp-1 functions as a transcriptional repressor that is critical in regulating the immune responses of these cells by promoting the presentation of antigens, secretion of cytokines, and maintenance of immune homeostasis [45,46]. The differentiation and activation state of macrophages is regulated by Blimp-1 in order to drive tissue-specific immune responses. These cells are allowed to integrate environmental signals and adjust their functions as a function of this inflammatory or autoimmune context. Further experimental studies reveal that Blimp-1 is required to limit the production of pro-inflammatory cytokines and to preserve immune homeostasis in mice and human macrophages. In Blimp-1 knockout models, for example, the secretion of cytokines, such as tumor necrosis factor-α (TNF-α), and macrophage activation are increased, resulting in exacerbated inflammatory responses [3,47]. This finding is particularly relevant in autoimmune disease, in which the dysregulation of macrophage activity is the cause of chronic inflammation.

Similar immune processes in dendritic cells are regulated by Blimp-1, demonstrated to influence the expression of genes for antigen presentation and cytokine production. Blimp-1 represses the transcription of pro-inflammatory genes to maintain balance in the immune response [48]. Blimp-1 knockout models of DCs have revealed enhanced DC activation as well as the increased production of inflammatory mediators in association with autoimmune diseases [49]. In addition, Blimp-1 affects the differentiation and function of monocytic-derived dendritic cells and macrophages in tissue [50]. Its role as a dynamic regulator permits the fine tuning of immune responses according to these environmental clues and thereby preserves tissue-specific immune responses during health and disease [35]. Together, these results suggest that Blimp-1 is required for the maintenance of DCs and macrophage immune homeostasis. The modulation of pro-inflammatory cytokine expression and the regulation of antigen presentation make this molecule important in autoimmunity as a means of preventing the overactivation of immune responses and maintaining balance in the inflammatory response.

### 2.4. Regulation in Other Cells

Moreover, Blimp-1 is a sine qua non for immune cell differentiation, as well as for epithelial cell differentiation and germ cell specification in vertebrate species. Blimp-1 plays an integral role in terminal differentiation in epithelial tissues, and it is necessary for proper organ formation, such as the mammary glands and sebaceous glands [51]. Epithelial-cell-specific Blimp-1-deficient animals have been shown to experience severe developmental abnormalities, resulting in neonatal growth and survival defects. For instance, Blimp-1 represses MHC class I pathway genes during neonatal intestinal epithelial cell development and immune tolerance and regulates gene expression, being critical for neonatal growth and metabolic homeostasis [52]. Blimp-1 is also critical for germ cell specification, particularly in primordial germ cell development. Blimp-1 knockout mice were found to be deficient in developing functional germ cells and exhibit early embryonic lethality, supporting the transcription factor’s involvement in early development [53]. Specifically, Blimp-1 works with AP2c and *PRDM14* to promote germ cell lineage commitment and also participates in guiding morphogenesis in the cardiac outflow tract and posterior forelimb development [54].

In addition to the above, Blimp-1’s regulation is seen in other multiple tissues, including mammary gland formation, with Blimp-1 promoting cell proliferation and polarization in luminal progenitors. Blimp-1 regulates sebocyte homeostasis, directly repressing c-Myc in sebocyte progenitors, indicating Blimp-1’s indispensability for terminal epidermal differentiation [55]. These findings indicate a conserved role for Blimp-1 in both physiological and pathological epithelial cell development, such as during dysregulated epithelial to mesenchymal transition, as observed in breast cancer cells. Finally, Blimp-1’s effect on non-immunity tissues, such as epithelial cells and germ cells, demonstrates the broad functional impact of Blimp-1 during development and differentiation. These non-hematopoietic roles are indicative of a versatile transcription factor, as Blimp-1 is involved in the regulation of such different developmental pathways in distinct cell types and tissues.

Blimp-1 is also expressed in other tissues and cell types, in addition to its roles in epithelial and germ cell development, indicating a broad functional impact. For example, Blimp-1 has been detected in the central nervous system, where it affects neural progenitor cell differentiation and may modulate particular neural subtypes during embryogenesis [56]. Moreover, in the skeletal muscle, Blimp-1 contributes to muscle fiber specification and regeneration [57]. Furthermore, Blimp-1 is becoming increasingly appreciated for its regulatory roles in oxidative stress and tissue repair pathways in non-immune cells. For instance, the expression of Blimp-1 in lung epithelial cells is linked to the regulatory function of inflammatory responses in injury [58]. This confirms its ability to act as a versatile transcriptional regulator, extending beyond immune contexts to diverse cellular and tissue-specific processes.

### 2.5. Molecular Mechanisms of Blimp-1 Regulation

At the molecular level, Blimp-1 is a critical transcriptional repressor that recruits a constellation of chromatin-modifying enzymes to a defined set of gene loci [46,59]. For example, Blimp-1 can recruit histone deacetylases (HDACs) and histone methyltransferases such as G9a to modulate the chromatin state and to repress gene transcription [46,59]. Previous studies have shown that Blimp-1 recruits HDAC1/2 to shut off transcription at target loci. In addition, the compound interacts with G9a histone methyltransferase, exploiting this enzyme’s ability to repress gene expression by changing the chromatin structure. Blimp-1’s broad transcriptional modulation over diverse cell types can be exerted due to this epigenetic modification [59,60]. Blimp-1 also interacts with interferon regulatory factor 4 (IRF4), which plays a role in immune cell differentiation, and with signal transducer and activator of transcription 3 (STAT3), which is important for several immune signaling pathways, to integrate intricate regulatory programs [36,37]. This interaction is critical to drive the differentiation of many immune cells, such as plasma cells and T cells [30,61]. Moreover, extracellular signal-regulated kinase (ERK) phosphorylation promotes Blimp-1 expression in plasma cells, and PI3K-triggered Blimp-1 activation regulates B cell selection and homeostasis. Previous studies also demonstrated that ERK-deficient B cells had difficulties in producing plasma cells and thus confirmed the requirement of ERK signaling for the transcriptional regulation of *Prdm1* (gene encoding Blimp-1) [62]. Importantly, ERK signaling is required for plasma cell differentiation, indicating that—contrary to the view that plasma cells are non-dividing, terminally differentiated cells—ERK signaling, previously thought to be associated with cell division, has a critical role in antibody-secreting plasma cell differentiation. In addition, the activation of ERKs by cytokines including interleukin-4 results in the induction of Blimp-1 expression for subsequent B cell differentiation to plasma cells, clearly illustrating the intricate role of ERK signaling in immune responses [63]. Furthermore, phosphoinositide 3-kinase (PI3K) signaling induces Blimp-1 expression and is central to the control of B cell selection and homeostasis [64]. PI3K signaling through the B cell receptor promotes terminal differentiation in part by the upregulation of Blimp-1, a transcription factor that interferes with the proliferation and survival of activated B cells, thereby limiting the proliferation of activated B cells. Abnormal Blimp-1 expression in Pten-deficient animals following the dysregulation of the PI3K pathway in part results in defective B cell selection, with an increased proportion of autoreactive B cells and splenomegaly. Together, these results emphasize the tight interdependence of PI3K signaling and Blimp-1 in directing B cells along both their terminal differentiative and early selection pathways. In conclusion, we find that Blimp-1 exerts roles in the immune system broadly and beyond. As a key player in immune homeostasis and tolerance, its critical functions lie in in regulating plasma cell differentiation, T cell responses, and innate immune cell activity. This includes its involvement outside immune tissues, further complicating its biological roles, making Blimp-1 a key factor not only in immune regulation but also in the search for potential therapies for diseases in which immune regulation and inflammation play important roles.

## 3. Oxidative Stress in Chronic Diseases

Oxidative stress plays a pivotal role in the pathogenesis of chronic inflammatory diseases, and its regulation occurs primarily through ROS [65,66]. ROS, such as hydrogen peroxide (H_2_O_2_), superoxide anions (O_2_·^−^), and hydroxyl radicals (·OH), are byproducts of cellular metabolism, particularly from the mitochondria. ROS are critical to cell signaling and homeostasis under normal physiological conditions [67]. However, when their production overwhelms the body’s antioxidant defenses, oxidative stress develops, leading to general cellular damage. This imbalance is a hallmark of many chronic diseases, including IBD, RA, and atherosclerosis [11,12,13,14,15,16]. The primary pathogenic mechanism is mitochondrial dysfunction induced by ROS. ROS are mainly formed by mitochondrial oxidative phosphorylation; excess glucose metabolism in the mitochondria, especially under diabetic conditions, increases NADH and FADH_2_ production [68]. The overproduction of these electrons leads to accumulation in the electron transport chain, causing excessive ROS formation, which makes a central contribution to oxidative stress in chronic inflammatory diseases. Lipid peroxidation, protein oxidation, and DNA damage represent the effects of ROS on cellular components. Lipid peroxidation, which ROS are known to initiate, causing damage to cell membranes, results in the loss of membrane integrity [69]. In addition, ROS are known to attack proteins, as well as nucleic acids, causing dysfunction in the cellular machinery and mutagenesis, respectively. These effects speed up the chronic disease process, increasing the inflammation further.

Two key signaling pathways are activated by ROS. The nuclear factor kappa B (NF-κB) signaling cascade is the primary pathway activated by ROS. Upstream activators of NF-κB and pro-inflammatory cytokines such as TNF-α and interleukins are associated with ROS [70,71]. Beyond this inflammation pathway, it has been associated with diseases like RA and IBD. In addition, the Nrf2 pathway is activated in reaction to oxidative stress to enhance the transcription of antioxidant enzymes [72]. ROS-induced NF-κB activation versus the Nrf2-mediated antioxidant response defines the degree of oxidative damage and inflammation.

The body’s primary defense against oxidative stress includes enzymes such as SOD, catalase, and glutathione peroxidase. Unfortunately, these systems are impaired by chronic diseases, which again accelerate ROS-induced damage. Therefore, metallic enzymes have been used to improve the scavenging of ROS, mimicking these antioxidant enzymes in order to provide a new therapeutic tool to treat inflammatory diseases. For example, Prasad et al. developed histidine-functionalized zinc oxide (ZnNPs-H) and copper-doped zinc oxide (ZnCuNPs-H) nanoparticles that mimicked the activity of SOD1 to counteract ROS in Parkinson’s disease. These nanoparticles show enhanced ROS scavenging, excellent biocompatibility, and the efficient targeting of mitochondria in neuronal cells. The restoration of cellular redox balance and protection against mitochondrial neurotoxic damage may constitute natural SOD1 functions, thus representing a neuroprotective approach for PD therapy. Additionally, Yao et al. developed manganese oxide (Mn_3_O_4_) nanoparticles as stable, multifunctional nanozymes that mimicked natural ROS-scavenging enzymes like superoxide dismutase and catalase [73]. These Mn_3_O_4_ nanozymes efficiently neutralize superoxide, hydrogen peroxide, and hydroxyl radicals, surpassing the ROS elimination performance of CeO_2_ nanozymes. Mn_3_O_4_ nanoparticles led to significantly reduced ROS levels and ROS-induced inflammation protection in both in vitro and in vivo models, positioning them as promising candidates to treat ROS-associated diseases, including cardiovascular disease and cancer. In sum, excess ROS generation overwhelms the natural antioxidant defenses, thereby causing oxidative stress at the molecular level. Consequently, this imbalance activates inflammatory pathways, causing cellular damage and worsening chronic diseases such as RA, IBD, and atherosclerosis. Oxidative stress regulation relies on the disruption of mitochondrial function and the subsequent activation of pathways such as NF-κB and Nrf2 [74].

### 3.1. Rheumatoid Arthritis

It is known that oxidative stress [11,75,76] plays a vital role in the pathogenesis of RA and significantly contributes to the progression of this autoimmune disorder. Persistent synovial joint inflammation in RA results in the generation of ROS, including O_2_^·−^ and H_2_O_2_ in excessive amounts. Not only do ROS contribute to local inflammation, but they also activate a variety of cell types within the synovium, including synovial fibroblasts and immune cells, stimulating the secretion of the pro-inflammatory cytokines including TNF-α, IL-1, and IL-6 [75,77]. Key players in recruiting and activating immune cells, such as macrophages and neutrophils, cytokines set in motion the cycle of inflammatory and tissue damage within the involved joints.

Hyperplasia of the synovium, giving rise to the formation of pannus, which is a proliferative tissue layer that invades and degrades cartilage and bone, is one of the characteristic outcomes of ROS overproduction in RA. Within the pannus, ROS also promote the activation of the NF-κB and mitogen-activated protein kinase (MAPK) pathways [78,79]. In addition to amplifying the inflammatory response, these pathways induce the production of matrix metalloproteinases (MMPs), enzymes that contribute to extracellular matrix component breakdown and other processes involved in cartilage and bone breakdown. In addition, oxidative stress causes damage to proteins, lipids, and DNA in the synovium, further exacerbating tissue damage and perpetuating the ever-continuing vicious cycle of inflammation and joint degradation.

The main reason for the excess of ROS in RA is associated with mitochondrial dysfunction [79,80]. Moreover, mitochondria are the main source of ROS, and they are increasingly damaged under chronic inflammatory conditions. The result is that the normal function of the mitochondrial electron transport chain is damaged, causing the accumulation of ROS both in the mitochondrial matrix and cytosol. In turn, mitochondrial ROS activate proapoptotic pathways and disable the function of mitochondrial permeability transition pores (mPTP), which exacerbates synovial cell death and tissue damage. In addition, mitochondrial damage at the site of inflammation in the RA joints is not only toxic to the local joints but also results in systemic effects, where these patients have a higher risk of developing cardiovascular diseases, a common comorbidity in RA patients.

In addition to their roles in local tissue destruction, ROS also modulate immune responses. ROS directly affect immune cell functions; T cell, macrophage, and neutrophil activity is changed. In other words, ROS can cause oxidative modifications, such as to cellular components in these immune cells, resulting in the activation of pro-inflammatory pathways; this subsequently perpetuates the chronic inflammatory response in RA. On the other hand, ROS induced by the fast ROS generation in RA can cause oxidative stress, which can modulate antigenicity, potentially leading to autoimmunogenicity, which may underlie the autoimmune component of RA [81,82]; the immune system can misidentify synovial tissue and start to attack the healthy synovial tissue.

Because of the central role of oxidative stress in RA, therapeutic approaches designed to decrease the levels of ROS have received considerable attention. The neutralization of ROS and protection against oxidative damage are both critically dependent upon antioxidants, both endogenous and exogenous. Some of these are enzymatic, like SOD and catalase, for example, which detoxify the ROS, and others are non-enzymatic, like vitamins C and E [83,84,85]. However, ROS signaling has proven to be complex in RA, which has hindered the rational development of antioxidant therapies. More recently, the specific targeting of mitochondrial dysfunction and ROS production has been investigated, and drugs that alter mitochondrial ROS production or promote mitochondrial biogenesis have been proven to have some effects in slowing RA-associated inflammation and joint damage [86,87,88]. Targeted therapies are also in development, including the use of nanoparticles to deliver antioxidants directly to inflamed joints in order to minimize the systemic side effects while maximizing the therapeutic efficacy [89]. In sum, oxidative stress is a key player in the pathogenesis of RA and its contribution to the perpetuation of inflammation, synovial hyperplasia, and joint destruction. In RA, ROS, mitochondrial dysfunction, and immune responses interact to create a self-perpetuating cycle of inflammation and tissue damage. Antioxidant therapies are promising, but further study is necessary to fully appreciate the complex actions of ROS in RA and to improve these therapies for clinical application.

### 3.2. Inflammatory Bowel Disease

Oxidative stress is an important factor in the pathogenesis of IBD, and ROS intensify the chronic inflammation of the mucosa of the intestine [13,14,15,90]. This results in the breakdown of the epithelial barrier, allowing increased bacterial invasion and an increased immune response, ensuring the perpetuation of the inflammatory process. In IBD, chronic inflammation causes the increased production of ROS, degrading the mucosal layer of the gastrointestinal tract, thereby facilitating bacterial invasion. The result is that immune responses are further exacerbated, as well as inflammation. Oxidative damage to such cellular components as lipids, proteins, and DNA is induced by this process, and this is a critical determinant of IBD progression [15]. Marked increases in oxidative stress indicators such as MDA and 8-hydroxy-2′-deoxyguanosine (8-OHdG) have consistently been associated with IBD [91,92]. Lipid peroxidation and DNA damage biomarkers correlate with disease severity, and these biomarkers indicate lipid peroxidation and DNA damage. Adverse effects on redox homeostasis result from the overproduction of intracellular ROS and further lead to cellular and tissue damage, associated with chronic inflammation in diseases such as IBD. Mitochondrial dysfunction is a primary source of ROS in IBD [93]. Oxidative phosphorylation by mitochondria is implicated in the production of excessive ROS, particularly in the colonic tissue. By activating macrophages and releasing pro-inflammatory cytokines such as TNF-α and IL-6, this perpetuates the cycle of inflammation. Oxidative stress is now recognized to be an important component of IBD, which has led to new therapeutic avenues that employ antioxidant therapies, such as the use of compounds like N-acetylcysteine, glutathione, and vitamin C, which can ameliorate oxidative damage and inflammation.

### 3.3. Asthma

Oxidative stress is an important feature of the pathogenesis of asthma and is involved in the development of airway hyperresponsiveness, inflammation, and airway tissue remodeling [17,94]. Endogenous and exogenous sources of ROS, like superoxide anion and hydrogen peroxide from air pollution, allergens, and inflammatory cells recruited to the lungs during an asthma attack, have been previously reported [95,96]. ROS damage the epithelial cells lining the airways, causing increases in permeability and the release of pro-inflammatory cytokines such as IL-6 and TNF-α, all of which facilitates the propagation of inflammation [97,98]. Such oxidative damage then further activates tissue remodeling, which leads to narrow airways and underlies the typical symptoms of asthma, such as wheezing and shortness of breath. Furthermore, various signaling pathways, including the MAPK and NF-κB pathways, can be activated by oxidative stress and play an important role in inflammation and consequent tissue damage [99,100]. Particulate matter (PM2.5) exposure has also been demonstrated to significantly worsen asthma symptoms by increasing the levels of oxidative stress, promoting ROS production, and decreasing antioxidant enzymatic activity [101,102]. The persistent inflammation that results from this imbalance between ROS production and the body’s antioxidant defenses is believed to play a role in airway remodeling and in asthma exacerbation. Consequently, antioxidants or therapies that regulate ROS production are targeted to prevent asthma progression.

### 3.4. Atherosclerosis

Oxidative stress is a very important factor in the pathogenesis of atherosclerosis and contributes to atherosclerotic plaque formation and instability within the arterial walls [16,103,104,105,106,107,108,109]. The overproduction of ROS, including O_2_^·−^, H_2_O_2_, and ·OH, starts this process. They are generated both from the mitochondrial respiratory chain and from NADPH oxidase [110,111]. Oxidative stress occurs when the levels of ROS exceed the capacity of endogenous antioxidant defenses, resulting in damage to vascular endothelial cells, the oxidation of low-density lipoprotein (LDL) cholesterol, and inflammation. The oxidation of LDL is an important early event in atherosclerotic lesion formation. The oxidation of LDL renders it more likely to be taken up by macrophages in the arterial wall, leading to the formation of foam cells, which typically form plaques in atherosclerosis [112,113]. The thickening of the arterial walls with the narrowing of blood vessels results from these foam cells, in combination with smooth muscle cell proliferation and inflammatory infiltrates. Plaques become unstable over time and contain oxidized lipids and cellular debris, which increases their risk of rupture. The rupture of plaques can activate pro-coagulant factors with the resulting formation of blood clots, which may lead to acute cardiovascular events, such as heart attacks or strokes.

In addition to promoting lipid oxidation, ROS play a key role in endothelial dysfunction, a hallmark of early atherosclerosis. Endothelial cells constitute the linings of blood vessels and are extremely sensitive to oxidative damage. With ROS elevation, endothelial cells become unable to adequately regulate vasodilation, primarily by decreasing the availability of nitric oxide (NO), a key vasodilator that endothelial cells produce [114,115]. Vascular inflammation is exacerbated by this dysfunction by recruiting immune cells such as monocytes and T cells to the arterial wall. These immune cells also release additional pro-inflammatory cytokines, such as IL 6 and TNF-α, which produces a self-replicating cycle of inflammation and oxidative damage.

In addition, oxidative stress can activate crucial signaling pathways in order to promote atherosclerosis. It is known that ROS activate the NF-κB and MAPK pathways, which regulate the expression of inflammatory genes and stimulate smooth muscle cell proliferation in the arterial wall [116,117]. This leads to thickened and stiffened arteries, part of the disease process. Furthermore, ROS can induce the apoptosis or programmed cell death of endothelial cells and macrophages inherent in plaques, thereby further destabilizing these plaques and increasing their likelihood of rupture. Exposure to environmental factors such as air pollutants like PM2.5 has been shown to contribute to the exacerbation of oxidative stress and promote the progression of atherosclerosis [118,119]. These pollutants increase ROS production, either directly by inducing cellular stress or indirectly by the attenuation of the antioxidant defense mechanism. This environmental contribution to the disease complicates the picture, illustrating the multifactorial nature of atherosclerosis.

Oxidative stress, characterized by the excessive production of ROS, plays a central role in the pathogenesis of atherosclerosis; thus, therapeutic interventions designed to decrease ROS levels are receiving significant attention. Studies of the neutralization of ROS and attenuation of oxidative damage by antioxidants including vitamins C and E have been performed [83,84,85]. However, while promising results have been achieved in clinical trials so far, these are mixed, because the signaling of ROS is a complex process; it can be both damaging and protective depending on the environment [120]. In recent years, novel therapeutic strategies, particularly the utilization of nanoparticle-based therapies to selectively scavenge ROS in atherosclerotic lesions, have emerged [121,122]. These nanoparticles are designed to specifically deliver antioxidant enzymes or ROS scavengers to the physiological site of oxidative damage, where they will reduce the local oxidative burden and consequently prevent plaque progression. Finally, ROS are central protagonists in driving lipid oxidation, endothelial dysfunction, and vascular inflammation in atherosclerosis. The rapid rate at which plaques form and destabilize due to an imbalance in ROS production and antioxidant defenses predisposes patients to cardiovascular events. Oxidative stress represents a target in efforts to slow down atherosclerosis through antioxidant therapy or ROS scavenging, but more research is needed to optimize the therapy and understand completely how ROS participate in the progression of the disease [123,124].

## 4. Blimp-1 Regulation in Antioxidant Responses

**Figure 1:**
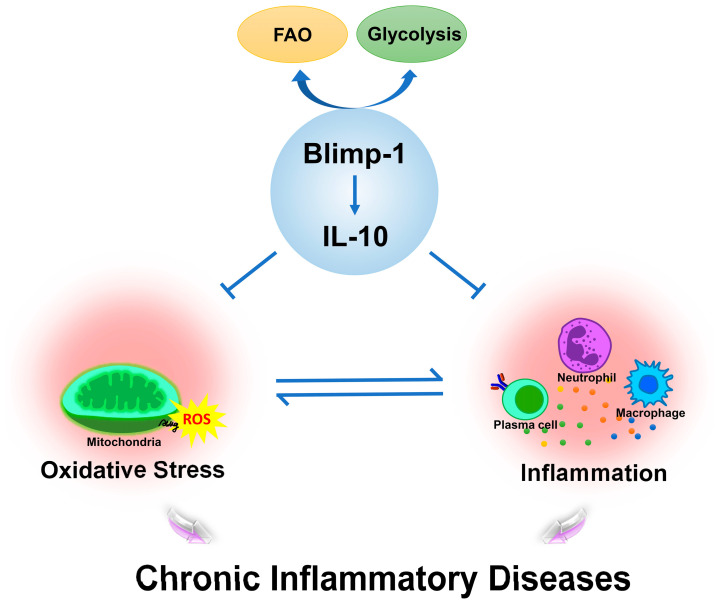
Role of Blimp-1 in regulating oxidative stress, metabolic reprogramming, and inflammation in chronic inflammatory diseases. This illustrates the role of Blimp-1 in regulating oxidative stress and inflammation, the two major pathogenic elements of chronic inflammatory diseases. The transcription factor Blimp-1 is required to balance FAO and glycolysis; increases IL-10 production, which may reduce excessive oxidation or ROS; and prevents an inflammatory response. Therapeutic interventions that involve targeting Blimp-1 pathways represent an approach aimed at reducing oxidative damage and inflammatory activity in chronic conditions, such as cardiovascular disease, diabetes, and autoimmune disorders.

The association of Blimp-1 with antioxidant responses, mediated in part through the regulation of IL-10 production, underlines a key function of this molecule in controlling inflammation and oxidative stress. Figure 1 illustrates the role of Blimp-1 in balancing oxidative stress and inflammation through IL-10 regulation, highlighting its potential as a therapeutic target for the management of chronic inflammatory diseases.

### 4.1. Blimp-1 Regulating IL-10 Production

Blimp-1 is a key transcriptional regulator of IL-10 production in Tregs, Th17 cells, and ILC2s. The modulation of immune responses and limiting inflammation-induced damage are well known in IL-10, an anti-inflammatory cytokine. As such, Blimp-1 regulates IL-10 to dampen pro-inflammatory responses and thus reduce the oxidative stress created by chronic inflammation, thereby maintaining systemic metabolic homeostasis.

**Regulation in Tregs:** Central to immune tolerance and preventing autoimmune diseases are Tregs [2]. Blimp-1 directly regulates IL-10 expression in Tregs, and this axis is required to suppress inflammation, particularly in metabolic tissues, such as the adipose tissue, where IL-10 is key in modulating insulin resistance and energy expenditure. Blimp-1 is required by adipose-tissue-resident regulatory T cells (aTregs) for their functional activity in preserving metabolic homeostasis [5]. The presence of Blimp-1 is necessary for the ability of aTregs to secrete IL-10 and consequently their effective suppression of inflammation and ability to prevent the beiging of white adipose tissue. The regulatory function of immune cells in the beiging process is regulated by Blimp-1, enhancing IL-10 production in aTregs to suppress this process [125,126,127]. In turn, IL-10 reduces oxidative stress by increasing the expression of antioxidant enzymes such as SOD, protecting tissues from inflammation-induced oxidative damage [126,128,129]. Therefore, Blimp-1, by using IL-10, serves as a critical intermediate between immune regulation and metabolic homeostasis, particularly in obese/insulin-resistant states.

**Regulation in Th17 Cells:** In the case of Th17 cells, which are pro-inflammatory and associated with autoimmune diseases like IBD, Blimp-1 makes an important contribution to the ability of these cells to transition from a pathogenic to a regulatory phenotype by promoting IL-10 gene production. The primary anti-inflammatory cytokine IL-10 suppresses the inflammatory activity of Th17 cells and thus lessens tissue damage and oxidative stress. It has been demonstrated that Blimp-1 is necessary for the production of IL-10 in Th17 cells. The activation of Blimp-1 is promoted by an upstream factor such as Ascl2, which induces Blimp-1 expression. For example, Ascl2 in Th17 cells was identified in a study in 2019, which showed that Blimp-1 is critical for the Ascl2-induced production of IL-10 [9]. Through this mechanism, Blimp-1 affects Th17 cell metabolism and function. IL-17A expression decreased but the production of the anti-inflammatory cytokine IL-10 increased in Th17 polarization conditions in CD4^+^ T cells overexpressing Ascl2. These data indicate that Ascl2 inhibits differentiation into Th17 cells and also promotes IL-10 production through the regulation of Blimp-1. It was found that Ascl2 induced Blimp-1 overexpression, and Blimp-1 was required for Ascl2 to promote IL-10 production. These findings establish a functional role for Blimp-1 in Ascl2-induced IL-10 production. On the other hand, the regulation of Th17 cells by Blimp-1 was additionally supported by a 2020 study with Sauchinone in IBD. Blimp-1 was also demonstrated to mediate the anti-inflammatory effects of Sauchinone via IL-10 production in Th17 cells [8]. A shift in the Th17 response from pathogenicity to regulatory, as a result of Blimp-1 expression, decreases pro-inflammatory cytokines and increases cells’ ability to mitigate inflammation-induced oxidative stress. A key factor in this Th17 cell maintenance of inflammatory homeostasis is represented by the Blimp-1/IL-10 axis, which promotes tissue repair, suppresses excessive immune responses, and regulates the metabolic balance in Th17 cells in inflammatory environments. Blimp-1 may protect against intestinal tissue damage due to ROS production and maintain immune homeostasis via an increase in IL-10 secretion, which may result in ROS reduction.

**Regulation in ILC2s:** ILC2s’ IL-10 production is also crucially dependent on Blimp-1 [21]. Blimp-1 is required for the induction of IL-10 from ILC2s, which mediate airway hyperreactivity and tissue repair; it is also required for the anti-inflammatory cytokine to combat inflammation and oxidative stress in airway diseases. ILC2s’ production of IL-10, regulated by Blimp-1, not only suppresses inflammation but also alters the metabolic pathways of ILC2s away from fatty acid to glycolytic metabolism. In turn, this metabolic reprogramming is essential for ILC2s to enhance their anti-inflammatory capacity and limit airway hyperreactivity and chronic inflammation, as seen in asthma conditions [130,131]. In addition, Blimp-1-mediated IL-10 production reduces ROS generation, which is crucial for the removal of oxidative stress and thereby protection from tissue damage.

### 4.2. IL-10’s Role in Antioxidant Defense

IL-10 is an essential anti-inflammatory cytokine with the capacity to regulate immune responses [22]. This is achieved by the prevention of the production of pro-inflammatory cytokines, but IL-10 also participates in oxidative stress removal [24,132,133]. In particular, in chronic inflammatory conditions, this is critical, since the excessive accumulation of ROS can aggravate tissue damage. Taken together, IL-10 mitigates ROS production and enhances the activity of antioxidant enzymes, thereby protecting cells from oxidative damage [23,134].

**Relationship between IL-10 and ROS**: ROS are naturally occurring byproducts of cellular metabolism, but, when their production exceeds the level of control, excessive ROS are produced. This is a hallmark of inflammation and oxidative stress, which occurs, for example, in chronic diseases such as RA and IBD. Free radicals such as superoxide anions (O_2_^·−^) and non-radical molecules such as H_2_O_2_ are ROS. In healthy conditions, a cell exhibits a balance among ROS production and elimination, which is mainly maintained by antioxidant systems. ROS production is excessive, however, in the case of chronic inflammation; as a result, DNA, proteins, and lipids are damaged. It also damages cellular components and perpetuates the inflammatory response. However, IL-10 limits the production of ROS in inflamed tissues that have been affected by harmful bacteria. IL-10 can lower ROS production by downregulating NADPH oxidase [135], an enzyme complex that generates ROS in immune cells such as macrophages. In addition, IL-10 modulates the activation of mitochondria to reduce pro-oxidative responses from excessive ROS release during inflammation.

**IL-10’s regulation of antioxidant enzymes:** ROS neutralization is important in protecting cells from oxidative stress, and antioxidant enzymes are crucial. SOD and glutathione peroxidase (GPx) are two key enzymes in this antioxidant defense system. SOD catalyzes the dismutation of superoxide anions into oxygen and hydrogen peroxide, and GPx accomplishes the reduction of H_2_O_2_ to water, with glutathione as a substrate. Studies have shown the increased expression and activity of SOD and GPx together with IL-10 in the antioxidant defense system [136,137]. In several models of inflammation, the IL-10-induced upregulation of these enzymes has been shown to result in a striking reduction in oxidative damage [22,23,24,132,133,134,135,136,137,138]. In cardiac myocytes exposed to oxidative stress, IL-10 was demonstrated to maintain the activity of SOD, catalase, and GPx to halt the buildup of detrimental ROS [134]. Blimp-1 overexpression alleviated vascular endothelial dysfunction and oxidative stress in DM-CHD rats, according to Chen et al. [10]. Treatment with lentivirus-transduced Blimp-1 significantly suppressed pro-oxidant MDA and enhanced the antioxidant SOD levels, indicating that Blimp-1 suppressed oxidative stress in DM-CHD. As a transcription factor, Blimp-1, which promotes IL-10 production, was identified as a possible key player in the regulation of SOD. Furthermore, these antioxidant enzymes are indirectly boosted by Blimp-1 by increasing their expression of IL-10, which serves as evidence of the relation between the functions of IL-10 as an immune regulator and an antioxidant.

**IL-10 protection in chronic inflammatory diseases:** IL-10 plays a particularly important protective role in chronic inflammatory diseases, in which ROS-driven oxidative stress is implicated in disease progression. Unchecked ROS production in such conditions as rheumatoid arthritis and IBD helps to destroy tissues and leads to further inflammation. The ability of IL-10 to inhibit ROS production and enhance antioxidant defenses renders IL-10 a key protective factor in these diseases [134,138]. Kaur et al. investigated the balance between TNF-α and IL-10 in mitigating oxidative-stress-induced cardiac injury [134]. TNF-α increased oxidative stress markers and cell injury in rat cardiac myocytes, while IL-10 alone showed no effect. However, IL-10 effectively countered TNF-α’s damaging effects, with optimal protection at an IL-10/TNF-α ratio near 1:1. Excessive TNF-α or high oxidative stress via H_2_O_2_ exacerbated cell damage. These results highlight that a balanced IL-10/TNF-α ratio is essential in protecting against oxidative stress in heart cells. In IBD, IL-10 negates the oxidative stress caused by pro-inflammatory cytokines such as TNF-α, preserving the benign functioning of the intestinal epithelium and sustaining gut barrier functional integrity [8]. Additionally, Carmen et al. assessed genetically modified lactic acid bacteria (GM-LAB) with anti-cancer properties in a colon cancer mouse model [137]. GM-LAB was engineered to produce antioxidant enzymes such as catalase or superoxide dismutase or the anti-inflammatory cytokine IL-10. Compared to wild strains, GM-LAB showed significant anti-cancer effects, particularly in mixtures targeting inflammation and oxidative stress. These findings suggest that GM-LAB mixtures could help to reduce the colorectal cancer risk in patients with chronic intestinal inflammation by creating a less inflammatory gut environment. This protective effect is critical in the prevention of IBD progression and complications such as colorectal cancer, in which chronic inflammation and oxidative stress are major contributing factors.

**IL-10 in other antioxidant mechanisms**: The direct regulation of antioxidant enzymes is only one of IL-10’s effects. In addition, it modulates other signaling pathways that are involved in antioxidant defense. For example, IL-10 might prevent the activation of the pro-inflammatory transcription factor NF-κB and its roles in the upregulation of pro-inflammatory cytokines and ROS-producing enzymes [139]. IL-10 lowers these inflammatory signals by preventing NF-κB activation and thus reducing oxidative stress. IL-10 has also been shown to restore the levels of other antioxidant molecules, like glutathione, an important intracellular antioxidant [22]. IL-10 increases the glutathione levels in models of sepsis, thus protecting cells from the oxidative damage traditionally seen in this condition [138]. These results suggest that IL-10 plays a multifaceted role in antioxidant defense, both enzymatic and non-enzymatic.

### 4.3. Integration of Blimp-1, IL-10, and Antioxidant Responses

To understand the multifaceted role of Blimp-1 in regulating IL-10, antioxidant responses, and metabolic reprogramming, we conducted a systematic review of key studies. The key studies included in Table 2 and Table 3 were selected through a rigorous process. Using the PubMed/NCBI database, a total of 83 papers were identified, with 28 papers retrieved using the keywords “Blimp1, inflammation”, 16 papers using “Blimp1, transplantation”, and 39 papers using “Blimp1, immune” in their titles and abstracts. From these, further screening was conducted to refine the selection. Two authors independently reviewed and double-checked the papers to identify studies specifically focusing on Blimp-1 and its role in regulating antioxidant responses, IL-10, or metabolic reprogramming. Ultimately, nine key studies were identified that specifically addressed the relationship between Blimp-1 and its regulation of oxidative stress and metabolic reprogramming in the context of inflammation. These studies formed the foundation for the data presented in the tables.

#### 4.3.1. Regulation of Blimp-1 in Antioxidant Responses in Chronic Diseases

**Diabetic coronary heart disease:** The effects of Blimp-1 on oxidative stress in DM-CHD, specifically with the regulation of Th9 cells and on oxidative stress markers, were studied directly [10]. Blimp-1 overexpression is proven to be effective in the reduction of oxidative stress, since the levels of MDA, a major signaling molecule of lipid peroxidation and cellular damage, are strongly related to atherosclerosis and coronary heart disease [143]. Moreover, increased activity of SOD, a major antioxidant enzyme responsible for the neutralization of superoxide anions [144,145], was seen, which indicated that Blimp-1 overexpression enhanced the antioxidant defense systems of the body. Blimp-1 protects against atherosclerotic plaque formation and the related deterioration in cardiovascular function by reducing both oxidative stress and inflammation. Blimp-1 is crucial to protect the cardiovascular system against oxidative injury in the context of diabetes, both by controlling the differentiation of Th9 cells and by increasing SOD activity in DM-CHD.

**Inflammatory bowel disease:** Short-chain fatty acids (SCFAs) such as butyrate regulate the expression of AREG in DCs via the GPR43 and Blimp-1 pathways [20,146,147]. A member of the epidermal growth factor (EGF) family, AREG is involved in maintaining intestinal homeostasis and in the inhibition of intestinal inflammation in IBD. The expression of Blimp-1 is critical in the regulation of this process, because butyrate-induced AREG expression protects against inflammation in mouse models of colitis [148,149]. Xiu et al.’s findings highlight the role of Blimp-1 in the protective anti-inflammatory and antioxidant responses mediated by DCs and the gut microbiota [20]. In DCs, Blimp-1 was identified as a critical transcription factor that promoted AREG expression induced by butyrate, a function that is critical in reducing intestinal inflammation [150,151]. The gut microbiota is responsible for the production of butyrate from the fermentation of dietary fiber, which has anti-inflammatory, antioxidant, and cellular-metabolism-modulating effects through the GPR43 signaling pathway. This relationship between the metabolic and antioxidant systems reveals the dual role of SCFAs in regulating both inflammation and oxidative damage in the gut. A study demonstrated that butyrate promoted Blimp-1-dependent AREG expression and that AREG was a critical factor induced during damage to promote repair, reduce oxidative stress, and restore function. The potential reduction of the inflammatory response and inflammation-induced oxidative damage by Tregs is also promoted by AREG expression by DCs. Blimp-1 modulates DC metabolism and functions to promote AREG expression; by extension, it supports DC factors in maintaining the gut oxidative stress balance. This analysis further indicates that the GPR43-mediated SCFA signaling pathway represents a major regulator of metabolic and antioxidant properties. The regulation of AREG expression mediated by Blimp-1 serves to reduce oxidative stress induced by gut inflammation. Future explorations into how Blimp-1 regulates the expression of additional antioxidant enzymes, such as SOD, could help to further protect against oxidative damage. Taken together, Blimp-1, regulated by SCFAs, enhances the expression of AREG, a factor that is necessary for inflammation-induced oxidative damage mitigation, particularly in models of intestinal inflammation. Blimp-1 is required in linking SCFA signaling to protective, anti-inflammatory, and antioxidant responses in the gut.

In summary, these studies collectively highlight the role of Blimp-1 in regulating anti-inflammatory and antioxidant functions in diverse pathologic situations. In these studies, Blimp-1 serves as a critical transcription factor to bridge cellular metabolism (SCFA signaling) and antioxidant responses (SOD activity and AREG expression) to protect the tissues during oxidative damage. Due to its role in integrating inflammatory and antioxidant pathways, it is also a target deserving further research in the management of chronic diseases.

#### 4.3.2. Regulation of Blimp-1 in IL-10-Mediated Antioxidant Responses in Chronic Diseases

**Asthma:** Blimp-1 has previously been found to be important for IL-10 production in ILC2s in airway inflammation models [21,130,131]. The regulation of oxidative stress and limitation of airway hyperreactivity require IL-10 production. Howard et al. demonstrated that the Blimp-1-induced metabolic reprogramming of ILC2s from fatty acid oxidation to glycolysis is essential for their anti-inflammatory function [152,153]. In this study, transcription factors such as Blimp-1 and c-Maf were demonstrated to be important in inducing IL-10 production by ILC2s [154,155], cells that are important in allergic inflammation and tissue repair. In addition to being an anti-inflammatory agent, IL-10 regulates ILC2 metabolism depending on Blimp-1 and shifts ILC2s’ energy production away from fatty acid oxidation and toward glycolysis. Importantly, this metabolic shift is necessary for ILC2s to exert their anti-inflammatory properties and restrict airway hyperreactivity and inflammation. Moreover, Blimp-1 controls ILC2 metabolism to instruct ILC2s to limit inflammation and initiate repair. This metabolic reprogramming allows ILC2s to shift into a more anti-inflammatory state, which is driven by fatty acid oxidation, to glycolysis through Blimp-1. Their dynamic control of immune cell metabolism indicates how Blimp-1, as a transcription factor, can modulate immune function and energy pathways that are critical in suppressing airway inflammation and hyperreactivity. Taken together, these findings indicate that Blimp-1 is a crucial transcription factor for the control of metabolic pathways in ILC2s, including the switch from fatty acid oxidation to glycolysis. Controlling inflammation and promoting tissue repair is essential and shows the close relationship between immune function and cellular metabolism in maintaining respiratory health. The regulation of this metabolic switch represents a key role for Blimp-1 in the dampening of the immune response and suppression of airway hyperreactivity, the intersection of which is linked to respiratory health.

**Inflammatory bowel disease:** Blimp-1 is required for IL-10-mediated antioxidant responses and is important in regulating immune cell metabolism and in the control of inflammation, including in IBD. Studies of the Ascl2–Blimp-1 axis suggest Blimp-1 as a critical regulator of IL-10 production in Th17 cells, which, if metabolically dysregulated, can promote the pathology seen in IBD [9]. The overexpression of Ascl2 results in increased Blimp-1 levels, enhanced IL-10 production, and decreased pathogenic activity of Th17 cells. The expression level of Ascl2 was significantly lower in the inflamed intestinal mucosae of IBD patients compared to healthy controls. Furthermore, Ascl2 expression was negatively correlated with the severity of the disease, and a marked decrease in Ascl2 expression in IBD patients was noted [156]. An analysis of Ascl2 expression in germfree mice indicated the elevated expression of Ascl2 with little microbiota suppression of Ascl2 expression. The gut microbiota regulates Ascl2; after gut microbiota removal via antibiotic treatment, the Ascl2 expression levels increased. The overexpression of Ascl2 attenuated colitis symptoms in mice by reducing inflammation and decreasing Th17 cell infiltration in Ascl2-overexpressing experimental colitis mouse models, revealing that Ascl2 is a potential therapeutic target for the treatment of colitis. It is important that this switch from a pro-inflammatory to an anti-inflammatory phenotype occurs to control oxidative stress and tissue damage. Blimp-1 works by inducing high IL-10 production, which is key to quelling out-of-control inflammatory responses; for this, energy metabolism must be redirected. The production of IL-10 correlates with the metabolic state of the cell, and Blimp-1’s function may be to regulate metabolic pathways to foster anti-inflammatory activity. The findings of these experiments suggest that Blimp-1 may additionally affect the metabolic programming of Th17 cells and serve to optimally divide energy distribution and support IL-10’s anti-inflammatory effects. By modulating these mechanisms, Blimp-1 also may serve as a facilitator of the antioxidant response in the intestines in order to help protect against inflammation-induced oxidative damage in the intestinal environment. On the other hand, IL-10 production by Th17 cells in the presence of the compound Sauchinone, which has anti-inflammatory and antioxidant properties, was studied, and the role of Blimp-1 in this was explored [8]. The study showed that Sauchinone increased IL-10 production via Blimp-1 in an IBD model and decreased inflammation in TNBS-induced IBD. IL-10-mediated antioxidant defense may indirectly be responsible for the Sauchinone-mediated reduction in the oxidative damage caused by oxidative stress.

**White adipose tissue beiging**: The role of Blimp-1 in the regulation of IL-10 is important in metabolic diseases such as obesity and insulin resistance [5]. Tregs in white adipose tissue (WAT) secrete IL-10 and suppress white adipocyte “beiging”, a pro-thermogenic process, through secreted IL-10 [125,126]. Mice lacking Blimp-1 selectively in Tregs displayed enhanced insulin sensitivity, decreased fat mass, and improved glucose tolerance, particularly under a high-fat diet, according to Beppu et al. [5]. This is caused by the fact that, in the absence of Blimp-1, Tregs synthesize less IL-10, which, in turn, results in the conversion of white adipose tissue into “beige” fat, such as a transformation to brown-like energy-consuming adipocytes. Using reduced Blimp-1 expression, this study showed increased uncoupling protein 1 (*UCP1*) and *PRDM16*—a key thermogenic gene—which increased the metabolic activity of the adipocytes [157,158]. The beiging process is suppressed by Treg-produced IL-10, and Blimp-1 is necessary in sustaining these levels of IL-10 [128,129]. This shows how Blimp-1 affects adipose tissue metabolism via IL-10 and participates in the equilibrium between pro- and anti-inflammatory responses in metabolic disease. Across disease contexts, these key studies demonstrate the multifaceted role of Blimp-1 in linking immune regulation, IL-10 production, and antioxidant responses. Here, we show that Blimp-1 regulates oxidative stress, cell differentiation, and metabolic pathways to be a crucial factor in inflammatory and metabolic diseases.

In summary, these studies show that Blimp-1 is a critical regulator of immune function and metabolism through its regulation of IL10 production and antioxidant responses. It governs metabolic shifts in ILC2s to prevent such inflammation in asthma. It supports anti-inflammatory Th17 cell activity and antioxidant defenses elicited by SCFAs in IBD. In WAT, it modulates the IL-10 levels and regulates fat metabolism and inflammation. These studies demonstrate Blimp-1’s central role in linking immune regulation, metabolism, and antioxidant responses across inflammatory and metabolic diseases. By modulating oxidative stress and inflammation, Blimp-1 becomes a key therapeutic target for various pathological conditions.

#### 4.3.3. Regulation of Blimp-1 in T Cells in Chronic Diseases

**Atherosclerosis:** Tissue-resident memory (TRM) T cells are a subset of T cells that persist in the tissue at the site of prior antigen recognition and protect the body against reencounter and prevent atherosclerosis. Specifically, TRM cells, marked by CD69 and CD49α, are present in atherosclerotic lesions and seem to contribute to lesion stability [141]. Xie et al. found that Blimp-1, along with Hobit (a Blimp-1 homolog in T cells), was a key transcription factor driving TRM cell formation [2,159,160]. TRM cells live in the tissue and do not circulate in the blood to deliver immune responses in the vicinity. In atherosclerotic plaques, TRM cells express a unique gene program characterized by high levels of Blimp-1. Maintaining these cells in the tissue, especially in inflammatory environments such as atherosclerotic plaques, depends upon this profile. While the above study does not deal with metabolism explicitly in cells, Blimp-1 may affect metabolism through its function in the regulation of TRM cell differentiation and retention. The metabolic demands of immune cells are altered in such inflammatory diseases as atherosclerosis, and Blimp-1 may regulate the cell survival and function of TRM cells in hypoxic and nutrient-poor environments, found in local lesions such as atherosclerotic plaques. TRM cells contribute to macrophage reduction in the lesion and promote plaque stability under the control of Blimp-1, suggesting that Blimp-1 could additionally promote cell metabolism, which favors tissue remodeling and stability. Blimp-1 deficiency decreased the number of TRM cells and paradoxically increased the number of plaque macrophages in mouse models; these are the very cell types associated with plaque instability. Therefore, this finding shows that Blimp-1 influences TRM cells’ fates in a manner that is also jointly responsible for supporting macrophage metabolic pathways that are known to dictate macrophage behavior and extracellular matrix integrity. Because of this, Blimp-1 is highlighted in this work as necessary for the formation and function of TRM cells within atherosclerotic lesions. In addition to regulating TRM-mediated immune responses, Blimp-1 may influence metabolic processes involved in cell survival in inflammatory environments, specifically stabilizing plaques and regulating macrophage content.

**Kidney rejection:** In chronic kidney rejection, chronic T-cell-mediated rejection (TCMR) is one of the major causes of kidney transplant failure, characterized by intimal thickening and alloimmune cell infiltration. Effector memory CD8^+^ T cells are believed to play a critical role in this chronic rejection process. Curci et al. demonstrated, through a gene expression analysis of kidney biopsy samples, that there is a distinct gene expression profile in chronic TCMR compared to acute TCMR [140]. The OX40 signaling pathway was significantly upregulated in chronic TCMR, and this pathway is associated with the generation of effector memory CD8^+^ T cells. Blimp-1 expression was also upregulated in chronic TCMR, and it is considered an important transcription factor for the differentiation of effector memory CD8^+^ T cells [39,44]. Other markers, such as KLRG-1 and CD25, which are markers of effector memory CD8^+^ T cells, displayed altered expression, supporting the involvement of effector memory CD8^+^ T cells in chronic TCMR [161,162]. According to this study, we speculate that Blimp-1 may function to regulate the metabolic activity of effector memory CD8^+^ T cells, which need specific metabolic support to maintain their long-term survival and function. Additionally, Blimp-1 may control glucose and fatty acid utilization pathways, including the expression of memetic genes in these cells. Consequently, Blimp-1 drives the formation and functionality of effector memory CD8^+^ T cells in chronic TCMR and may additionally influence their survival and functioning by modulating metabolic pathways, worsening chronic rejection.

**Skin rejection:** In fact, Wang et al. showed that Blimp-1 is important for the T cell differentiation of Tregs [6]. These are Tregs that help to keep the immune system in check, especially in the context of transplantation. In nature, anti-inflammatory Tregs are promoted by the overexpression of Blimp-1, which also suppresses pro-inflammatory cells such as Th1 cells [41,163]. A study also shows that Blimp-1 raises the levels of IL-10, a critical anti-inflammatory cytokine [40,41]. IL-10 is involved in metabolic reprogramming to support T cell functions that are essential in preserving immune homeostasis and dampening inflammation [21]. Consequently, we hypothesize that Blimp-1 may be involved in modulating metabolic programming in these T cells. Blimp-1 is likely involved in reprogramming Treg metabolism to allow these cells to survive in inflammatory environments. This can be quite important in cases like allograft rejection, where immune cells must survive in a nutrient-starved and hypoxic environment.

In summary, these studies suggest that Blimp-1 is a central transcription factor that connects immune regulation and metabolic processes. The survival and function of T cells (TRM cells, effector memory CD8^+^ T cells, and Tregs) in inflammatory and nutrient-deprived environments is assured. Blimp-1 stabilizes plaques in atherosclerosis by promoting TRM cell retention and reducing macrophage-induced instability. It is crucial for effector memory CD8^+^ T cells’ function and survival and thereby contributes to chronic kidney rejection. In skin rejection, it is needed for Tregs and functions to counteract inflammation and maintain immune balance. Consequently, Blimp-1 could have multifaceted action in chronic diseases through the regulation of T cell differentiation and function and metabolism. Being interconnected with metabolic pathways, it thereby represents a possible therapeutic target for the improvement of inflammatory, immune-mediated conditions.

## 5. Blimp-1 Regulation in Metabolism

### 5.1. Metabolic Reprogramming in Chronic Diseases

The reprogramming of metabolism, including switching between glycolysis and fatty acid oxidation (FAO), is critical to the progression and regulation of chronic inflammatory diseases, including RA, IBD, and atherosclerosis. This dynamic metabolic adaptation influences immune cell function, inflammation, and tissue homeostasis, which are critical to the pathogenesis and progression of these conditions.

#### 5.1.1. Glycolysis in Chronic Diseases

Glycolysis is an oxygen-independent, rapid pathway for the production of ATP, able to support cellular energy needs during acute immune responses. In chronic inflammatory diseases, however, glycolysis frequently becomes dysregulated and can continue to support a pro-inflammatory environment. In RA, cells including macrophages and synovial fibroblasts exhibit a metabolic shift to glycolysis despite oxygen availability (Warburg effect) [164,165,166]. This appears to drive inflammatory cytokine production, such as TNF-α and IL-6, in a glycolytic state conducive to synovial hyperplasia, joint destruction, and chronic inflammation [167]. In RA, the expansion of pathogenic T cells is also fueled by glycolysis [168]. Glycolytic reprogramming was reported in epithelial cells and in infiltrating immune cells in the inflamed intestinal mucosa of IBD [169]. Pro-inflammatory Th17 cells and neutrophils require glycolysis for survival and function in the gut, amplifying tissue damage and perpetuating inflammation [170]. In atherosclerosis, macrophages in atherosclerotic plaques become glycolytic [171,172]. This metabolic switch allows pro-inflammatory cytokine, ROS, and matrix-degrading enzyme production to be sustained, a process that results in plaque instability and vascular injury [173,174].

#### 5.1.2. Fatty Acid Oxidation in Chronic Diseases

Under normal or non-inflammatory conditions, FAO is a more energy-efficient metabolic pathway that is typically coupled to anti-inflammatory responses and cellular maintenance. The dysregulation of FAO can, in fact, significantly impact disease progression. In RA, Tregs and macrophages lose FAO, resulting in reduced anti-inflammatory capacity [175]. FAO in these cells has been shown to be restored, leading to reduced inflammation and tissue repair, indicating a potential therapeutic target [175]. In IBD, FAO is essential for intestinal barrier integrity and maintaining the epithelial cells’ energy supply. In IBD, the shift away from FAO to glycolysis contributes to epithelial cell dysfunction, increased permeability, and increased susceptibility to bacterial translocation and propagates inflammation [176]. In atherosclerosis, FAO in vascular smooth muscle cells and macrophages is essential in maintaining the cellular energy balance and anti-inflammatory activity. A reduction in FAO promotes lipid accumulation, foam cell formation, and the perpetuation of inflammation in atherosclerotic lesions [177].

The regulation of immune cell function and inflammation relies on the coordination of glycolysis and FAO. These metabolic pathways are often out of balance in chronic inflammatory diseases. Metabolic reprogramming utilizing glycolysis and FAO is a fundamental regulator of immune cell behavior and inflammatory responses in chronic inflammatory diseases. These metabolic shifts could be understood and targeted as a means to develop alternative treatment methods for inflammation and tissue damage.

### 5.2. Metabolic Impact on ROS and IL-10

#### 5.2.1. Effects of Glycolysis on ROS Production

As a central metabolic pathway, glycolysis is the flow of glucose anaerobically to ATP in the cytoplasm. Compared to mitochondrial OXPHOS, glycolysis produces less energy but more rapidly and at lower levels of ROS production. The Warburg effect refers to cancer cells and other proliferative immune cell types showing a preference for glycolysis, even in the presence of oxygen, a condition in which one would expect the oxidative phosphorylation pathway to be favored due to the abundance of oxygen [178]. ROS production in glycolysis is relatively limited, because ROS occur mainly outside the mitochondria. However, the byproducts of glycolysis fuel other metabolic pathways that produce large amounts of ROS. For example, pyruvate derived from glycolysis is shunted to acetyl-CoA, a TCA cycle intermediate in the mitochondria, where high levels of ROS are generated from leaky electrons from complexes I and III of the ETC [110]. A major source of superoxide anions, precursors of H_2_O_2_ (milder ROS involved in signaling at low concentrations, being harmful at high concentrations), is this leakage. Glycolysis is a means to limit oxidative stress by guiding glucose metabolites toward the pentose phosphate pathway (PPP) [179]. This generates NADPH, a reducing agent that is an important player in regenerating glutathione (GSH), the most potent of the cell’s antioxidants. This metabolic reprogramming enhances the antioxidant capacity of cells to prevent ROS-induced damage and enable cells to counter oxidative stress. This pathway is often upregulated by cancer cells, thereby maintaining a fine balance between proliferation and oxidative stress. Meanwhile, under metabolic stress, e.g., glucose deprivation, cells might switch to mitochondrial OXPHOS, which generates more ATP per molecule of glucose but also more ROS [180]. The excessive production of ROS may consequently result in oxidative damage, activating the cellular pathways for cell death should the antioxidant defenses be inadequate.

#### 5.2.2. Effects of Fatty Acid Oxidation on ROS Production

One source of ROS production is FAO, a vast metabolic pathway that is particularly present in mitochondria. The breakdown of fatty acids leads to acetyl-CoA, which enters the TCA cycle, potentiating mitochondrial respiration and leading to increased ROS generation [110]. This process is tightly regulated and is critically involved in energy homeostasis, particularly during times of low glucose availability. When electrons are transferred through the mitochondrial ETC, especially during FAO, there is increased production of superoxide radicals as a byproduct. In particular, complex II (succinate dehydrogenase) of the ETC is of critical importance, as it is in FAO ROS generation. An increase in FAO may enhance mitochondrial ROS production as the result of an increase in electron leakage from both complex I and complex II [110]. This is interesting as FAO exhibits the biphase regulation of ROS levels [181]. On the other hand, the contribution to ROS generation is accompanied by enhanced mitochondrial respiration. However, FAO supports the production of NADPH, needed for antioxidant defense. In a number of different pathological conditions, such as heart failure and cancer, this relationship between FAO and ROS has been observed. Augmented FAO in heart disease is associated with the enhanced production of ROS, which contributes to cardiac dysfunction. Oxidative stress in cells dependent on FAO for energy production has been correlated with cancer [181]. The levels of NADPH required for antioxidant defense and other cellular functions in some cancer cells are sustained by FAO [110]. For example, the inhibition of FAO in tumor cells results in increased ROS accumulation and cell death, indicating the importance of this pathway in redox balance management [181]. As such, FAO is a potential therapeutic target in preventing the metabolic adaptation of cancer cells and inducing stress in cancer cells to create oxidative stress. Furthermore, FAO-derived ROS contribute to immune modulation. For example, the inhibition of FAO in macrophages promotes their antimicrobial function by increasing the ROS production of these cells. These findings suggest that FAO may be an avenue by which to modulate immune cell ROS levels. Finally, glycolysis and fatty acid oxidation are both critical for ROS production, but by different mechanisms. Glycolysis, especially in the context of the Warburg effect, limits ROS production by shifting energy metabolism away from mitochondria. In contrast, the expression of mitochondrial OXPHOS or FAO increases ROS production significantly as a consequence of electron leakage in the ETC. The tight control of the balance between ROS formation and scavenging is essential in maintaining normal levels of various cellular phenomena, including cancer, cardiovascular diseases, and inflammatory diseases. The metabolic regulation of ROS provides meaningful insights into therapeutic strategies for diseases resulting from oxidative stress.

#### 5.2.3. Effects of Glycolysis on IL-10

Metabolic regulation is a major regulator of the immune system, and, in particular, glycolysis has been shown to regulate the production of IL-10, an important anti-inflammatory cytokine [21]. Metabolism to glycolysis was also shifted in macrophages and DCs when these cells encountered stimuli such as LPS. They result in metabolic reprogramming with the generation of specific metabolites, including citrate and succinate, which contribute to modulating cytokine gene expression. Importantly, the pyruvate kinase isozyme M2 (PKM2) is activated by LPS, which can regulate metabolism. PKM2 has been demonstrated to suppress elevated succinate and glycolytic reprogramming, enhance IL-10 production, and decrease IL-1β, a pro-inflammatory cytokine [182].

Glycolysis not only impacts pro-inflammatory cytokine production but also regulates IL-10 secretion through its effects on cellular energy homeostasis and immune response modulation. A key reduction in IL-10 production through blocking or reprogramming glycolysis in some inflammatory conditions indicates that IL-10 regulation is tightly coupled with glycolytic activity [183]. For example, the metabolic balance between glycolysis and OXPHOS in macrophages influences whether they become pro-inflammatory or anti-inflammatory [184]. Similarly, Th17 cell studies found that the inhibition of glycolysis increased IL-10 production, whereas the secretion of pro-inflammatory cytokines was reduced [185]. Regarding mycobacterium tuberculosis infection, the inhibition of the glycolytic shift in macrophages induces higher IL-10 and diminished IL-1β secretion [186]. This indicates that glycolysis participates in a tightly regulated balance between pro-inflammatory and anti-inflammatory responses, and the expression of IL-10 can be suppressed as part of this balance, with a shift toward glycolysis, except when tightly regulated by factors like PKM2.

In addition, metabolic pathways were found to augment the production of IL-10 in DCs by a variety of factors. For instance, butyrate, lactic acid, and adenosine, as well as ATP, have been found to be modulators that allow for the production of IL-10 in DCs [187,188,189]. Furthermore, the activation of the mammalian target of rapamycin (mTOR), a major regulator of cellular metabolism, has been shown to stimulate IL-10 production in DCs [190,191]; indeed, mTOR regulates signaling from nutrients and environmental stimuli to modulate metabolic pathways and regulate immune responses and thus represents a major regulator of IL-10 production. Adiponectin is another important factor in increasing IL-10 production through the AMPK pathway, which underlines the role of energy metabolism in regulating immune function [192]. Importantly, these findings demonstrate that glycolysis and the associated metabolic regulators are context-dependent and can either promote or inhibit IL-10 production.

#### 5.2.4. Effects of Fatty Acid Oxidation on IL-10

Fatty acid oxidation has a similar influence on IL-10 production in both T cells and macrophages, in addition to glycolysis. An example of this regulation is key during wound healing. During activation by efferocytosis, like the engulfment of apoptotic cells, macrophages may change their metabolism to favor the oxidation of fatty acids, leading to increased IL-10 production. The SIRTUIN1 signaling cascade is initiated through mitochondrial respiration, which drives the transcription factor PBX1 and promotes IL-10 production [193]. As such, investigations of the ways in which FAO regulates IL-10 secretion have gained attention recently. In fact, FAO stimulates OXPHOS, which is highly associated with IL-10 synthesis, especially in anti-inflammatory macrophages [184]. FAO, leading to the accumulation of long-chain fatty acids and thus boosting mitochondrial β-oxidation, is regulated during immune responses such as efferocytosis. It has recently been shown to increase the production of the anti-inflammatory IL-10, promoting the resolution of inflammation and tissue repair [193]. Furthermore, specific immune cell subsets are impacted differently by FAO, including ILC2s, in which a shift in metabolism from FAO to glycolysis resulted in increased IL-10 production [21]. In experimental models of colitis, the chemical inhibition of the nicotinamide phosphoribosyltransferase (NAMPT) enzyme, which is involved in NAD^+^ biosynthesis, has been shown to promote the polarization of monocytes and macrophages toward an IL-10-producing phenotype. This indicates that fatty acid oxidation and NAD^+^-dependent metabolic pathways play important roles in determining the anti-inflammatory profiles of immune cells [194].

Fatty acid oxidation has also recently been shown to influence the IL-10 production of T cells. Luu et al. show that SCFAs, like pentanoate, produced by the gut microbiota, can induce IL-10 in a variety of immune cell types, including T cells and regulatory B cells [185]. IL-10 production is enhanced by pentanoate through the reprogramming of metabolic–epigenetic interactions, stimulating glucose oxidation concomitantly with Th17 cell population suppression [185]. Moreover, in the same study, pentanoate was found to increase IL-10 gene expression in regulatory B cells, supporting a role for fatty acid metabolism in the fine tuning of anti-inflammatory responses. Gut-microbiota-derived SCFAs also act to maintain intestinal homeostasis by inducing IL-10 production in Th1 cells. The activation of the STAT3 and mTOR signaling pathways leads to the upregulation of the transcription factor Blimp-1, a key regulator of IL-10 gene expression [147]. This mechanism highlights the dependence of the systemic immune response on gut-derived metabolites by metabolic reprogramming. These findings provide evidence for the important role of metabolic pathways in the control of the balance between pro-inflammatory and anti-inflammatory cytokine production and suggest that fatty acid oxidation affects IL-10 production. The capacity to enhance IL-10 production while simultaneously reducing pro-inflammatory responses is critical in sustaining the balance of the immune system and avoiding excessive inflammation. Finally, glycolysis and fatty acid oxidation are both important metabolic pathways participating in the production of IL-10. Glycolytic reprogramming and metabolic shifts inside glycolytic immune cells can be adjusted to regulate the balance of cytokines, whereby specific metabolites, PKM2, and mTOR can further modulate the production of IL-10. Fatty acid oxidation and NAD^+^-dependent processing are implicated in counteracting inflammation and promoting IL-10 production by macrophages, T cells, and regulatory B cells. Studies of these metabolic influences offer insights into immune regulation and potential therapies for inflammatory diseases.

### 5.3. Integration of Blimp-1, IL-10, and Metabolism

**Asthma:** ROS are chemically reactive molecules with oxygen, and they have dual functions in biological systems [195,196]. At moderate levels, ROS have important signaling functions. However, when overly produced, most often during inflammatory responses, ROS contribute to oxidative stress and harm DNA, proteins, and lipids [197]. The acute and chronic production of excessive ROS exacerbates tissue injury and inflammatory responses in the lungs, especially in conditions such as asthma and other inflammatory respiratory diseases, contributing to disease progression [179]. ILC2s usually depend on FAO for energy production, predominantly under normal pro-inflammatory conditions, which is a process that produces increased levels of ROS via increased mitochondrial oxidative phosphorylation [110,153]. Nevertheless, the metabolic program of ILC2s is switched by Blimp-1 from FAO to glycolysis, and this process may drastically decrease ROS production [198]. Indeed, because glycolysis takes place in the cytoplasm and does not require mitochondrial oxidative phosphorylation, fewer ROS as generated. Howard et al. find that this metabolic shift is supportive of the production of the anti-inflammatory cytokine IL-10 [21]. Moreover, IL-10 has been proven to reduce the ROS levels and oxidative stress [22]. The protective role of Blimp-1 is particularly critical in chronic respiratory diseases involving oxidative stress as a key driver of disease progression. In these conditions, Blimp-1 functions as a fine-tuning factor that maintains the immune response and suppresses ROS generation while increasing the antioxidant defenses.

**Antibody-secreting cells:** Orchestrating plasma cell differentiation and driving metabolic reprogramming are critical functions of Blimp-1. More specifically, this transcription factor has been found to be required for B cells to transition to antibody-secreting cells (ASCs) and to drive metabolic processes including OXPHOS for the maintenance of the high energetic and biosynthetic needs in ASCs [142]. Blimp-1 acts during B cell differentiation by regulating the expression of a B cell identity suppression gene expression program coupled with the promotion of the ASC phenotype. Scharer et al. demonstrated that this transition is one of the essential features and includes metabolic reprogramming compatible with ASC formation and cellular proliferation [142]. This reprogramming, characterized by the major OXPHOS pathway, which is the most energy-efficient pathway for ATP generation, is required to support the energetically wasteful process of antibody production and secretion [199]. In a study of Blimp-1-dependent processes, it was observed that activated B cells (actB cells) undergo a series of cell divisions before committing to differentiation [142]. Notably, Blimp-1 is dispensable during the early proliferative stages of B cell activation but becomes critical at later stages, particularly by division 8, where the transcription factor’s role is indispensable for ASC differentiation. Cells lacking Blimp-1 failed to activate crucial metabolic genes associated with OXPHOS, such as isocitrate dehydrogenase 3 alpha (Idh3a) and lactate dehydrogenase A (Ldha), both of which are involved in the tricarboxylic acid (TCA) cycle and lactate production.

The shift to OXPHOS is a vital aspect of the Blimp-1-driven reprogramming process. In the absence of Blimp-1, cells show a failure to induce key components of OXPHOS, which are necessary for ASCs to meet their increased bioenergetic demands. However, cells do not differentiate into functional ASCs as a result of this failure. In the case of plasma cells, the OXPHOS pathway is especially important because it enables long-term survival and high rates of antibody secretion. Cells destined for ASCs also upregulate genes associated with the endoplasmic reticulum (ER) stress response, such as X-box binding protein 1 (Xbp1), another key target of Blimp-1 that is essential in managing the stress imposed by the large-scale production of immunoglobulins [35]. In contrast, cells that follow the non-ASC branch, characterized by a lack of Blimp-1 activity, fail to downregulate inflammatory response genes and are marked by diminished OXPHOS activity. These cells usually stay responsive to inflammatory signals, instead of proceeding to differentiate into ASCs. The bifurcation observed at this stage of B cell differentiation suggests that metabolic reprogramming, particularly the activation of OXPHOS, is a key determinant of whether a B cell becomes an ASC.

**White adipose tissue beiging:** A key regulator of aTregs, the transcription factor Blimp-1 is known as an important orchestrater of metabolic processes, particularly with regard to fatty acid oxidation and energy homeostasis. aTregs expressing Blimp-1 secrete the anti-inflammatory cytokine IL-10, which has important effects on adipose tissue metabolism, including the suppression of WAT browning [5]. Such inhibition has downstream implications for metabolic health, such as the regulation of fatty acid oxidation. In the context of metabolic reprogramming, Blimp-1 also affects glucose metabolism, in addition to fatty acid oxidation. Research demonstrates that the loss of Blimp-1 expression in aTregs results in a metabolic shift toward increased glucose oxidation rather than fat oxidation. In particular, IL-10 produced by Blimp-1-expressing aTregs appears to directly suppress the beiging of white adipocytes, a process associated with higher energy expenditure and enhanced glucose oxidation in beige adipocytes [125,126]. Maintaining energy homeostasis, this regulatory mechanism can also lead to metabolic dysfunction if disturbed. In an experiment where Blimp-1-deficient mice specific for aTregs displayed increased glucose tolerance and insulin sensitivity, with no marked changes in overall energy expenditure and food intake, this shift in metabolic preference from fatty acid oxidation to glucose oxidation was observed [200]. In these mice, the increased glucose oxidation appeared to stem from the elevated expression of thermogenic genes such as *Ucp1* and *Prdm16* in the adipocytes, which are typically associated with the beiging of WAT [158]. As such, the loss of Blimp-1 in aTregs promotes a metabolic state characterized by increased glucose usage, which may offer protection against diet-induced obesity (DIO) and insulin resistance. Beppu et al. demonstrated that Blimp-1 plays a complex role in regulating fatty acid oxidation by suppressing thermogenic processes in the white adipose tissue. In aTregs, Blimp-1 indirectly influences fatty acid oxidation by regulating IL-10 production, impacting the adipocytes’ metabolic activity [5,128]. The suppression of adipocyte beiging by Blimp-1 ensures that WAT is used for thermogenesis rather than storing fat. This mechanism is especially important in conditions like obesity, when fat accumulation is not in balance with fat expenditure, leading to further metabolic problems. It was found that, without Blimp-1, aTregs are unable to produce enough IL-10, resulting in enhanced WAT beiging. The advantages of this metabolic reprogramming include the prevention of excessive fat accumulation and the promotion of metabolic health under high-fat diet conditions. However, under normal conditions, Blimp-1 keeps this process under tight regulative control to avoid unnecessary fat oxidation and to conserve adipose tissue homeostasis.

In summary, these studies implicate Blimp-1 as a key transcriptional effector that integrates metabolism, immune regulation, and antioxidant responses. In asthma, it reduces oxidative stress by metabolic switching and IL-10 production. In ASCs, it is required for OXPHOS to provide the energy required for robust antibody secretion. Through IL-10 production, it regulates fatty acid oxidation and glucose metabolism and maintains adipose tissue homeostasis in WAT. Thus, Blimp-1 bridges immune responses, metabolic reprogramming, and the regulation of oxidative stress and chronic disease. As a regulator of metabolism, it is also positioned to play a role in tailoring metabolism to the functional needs of immune cells and tissues; therefore, its targeting may also represent a strategy for intervention in inflammation, respiratory diseases, and metabolic disorders.

## 6. Discussion

Excessive oxidative stress and metabolic dysregulation are often associated with chronic inflammatory diseases, like diabetes, cardiovascular disease, and autoimmune disorders. The transcriptional repressor Blimp-1 has emerged as a key regulator of immune function and cellular metabolism that integrates inflammatory responses and the antioxidant program. With the continued observation of Blimp-1’s role in a variety of immune cells and the ability to modulate oxidative stress and inflammation through this transcription factor, there is hope for the development of a therapeutic avenue aimed at Blimp-1. This section will survey the therapeutic potential of targeting Blimp-1 as an antioxidant/anti-inflammatory strategy, its clinical significance in treating chronic diseases, and the future directions of its research and application.

### 6.1. Therapeutic Potential of Blimp-1 as a Target

Since Blimp-1 is involved in regulating immune cell metabolism, anti-inflammatory cytokine production, and antioxidant responses, its in vivo potential as a therapeutic target is clear. Blimp-1 is a transcriptional repressor that is able to modulate these programs; given that chronic diseases like type 2 diabetes, cardiovascular disease, and autoimmune disorders are often driven by chronic inflammatory and oxidative stress, the ability to regulate Blimp-1’s activity would improve the body’s ability to offset these processes [5,6,8,9,10,20,21,140,141].

**Regulation of antioxidants**: A significant way in which Blimp-1 could be leveraged therapeutically is through its effects on the antioxidant response. Through the activation of genes encoding key antioxidant enzymes such as SOD, which scavenge the ROS that cause oxidative damage in tissues, Blimp-1 contributes to the cell’s ability to neutralize ROS [8,10]. Excessive ROS production in the setting of diabetes and cardiovascular disease contributes to the progression of the disease through endothelial dysfunction, inflammation, and tissue damage. If successful, targeting Blimp-1 might ultimately offer a way to enhance the antioxidant defenses of immune cells, especially those that contribute to chronic inflammation and are burdened by oxidative stress.

**Regulation of inflammation**: The central role of Blimp-1 in regulating anti-inflammatory cytokine production, particularly IL-10, has been emphasized [5,8,9,10,21]. IL-10 is well known for suppressing pathways that stimulate pro-inflammatory processes and mitigating immune-mediated tissue damage. Therapeutic strategies to upregulate Blimp-1 might increase IL-10 production via the most critical players in maintaining immune homeostasis, such as Tregs, Th17 cells, and ILC2s. Additionally, IL-10 has been shown to possess antioxidant potential, inhibiting ROS generation and promoting the production of antioxidant enzymes [22]. Blimp-1 may be a pivotal target for the modulation of the balance between pro- and anti-inflammatory responses, thereby dampening the chronic inflammation that characterizes diseases such as rheumatoid arthritis, inflammatory bowel disease, and asthma.

**Regulation of metabolic reprogramming:** Blimp-1 also regulates antioxidant and anti-inflammatory pathways but is strongly implicated in the metabolic reprogramming of immune cells [21]. Disruptions in cellular metabolism are frequently found in the setting of many chronic diseases, such as asthma, and often exacerbate oxidative stress and inflammation. Blimp-1 influences metabolic processes like fatty acid oxidation and glycolysis, fine tuning the immune response, and both suppressing excessive ROS production and promoting tissue repair. Thus, targeting Blimp-1 could aid in correcting the metabolic imbalances that occur in chronic diseases and hence also improve the control of inflammation and oxidative damage.

### 6.2. Clinical Implications: Treating Chronic Diseases by Targeting Blimp-1

Targeting Blimp-1 is clinically significant because it modulates both metabolic and immune pathways, being a versatile target for the treatment of an array of chronic diseases. Therapies that utilize Blimp-1’s regulatory functions, with the goal of treating the oxidative stress, inflammation, and metabolic dysfunction that are at the root of disease progression in diabetes, cardiovascular disease, and autoimmune disorders, would unify these fields.

**Figure 2:**
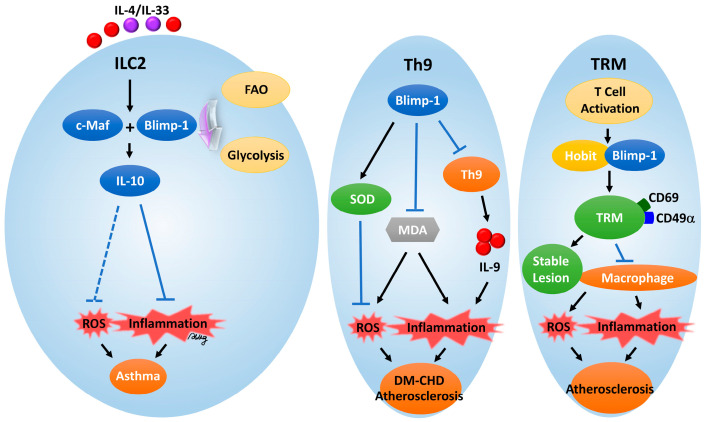
Blimp-1’s regulation of ROS and inflammation across immune cell types in chronic disease context. In immune cells such as ILC2s [21], Th9 cells [10], and tissue-resident memory T cells (TRM) [141], the contribution of Blimp-1 in the modulation of chronic disease-related oxidative stress (ROS) and inflammation is illustrated. In ILC2s, the induction of Blimp-1 and c-Maf induces IL-10 to mitigate asthma. Additionally, IL-10 has the potential to reduce ROS. Blimp-1 in naïve T cells induces superoxide dismutase (SOD) activity, which suppresses the oxidative stress marker MDA, inhibits Th9 differentiation, and alleviates inflammatory conditions related to diabetes mellitus–coronary heart disease (DM-CHD) and atherosclerosis. In TRM cells, Blimp-1 cooperates with Hobit to regulate macrophage activity and inflammation and to stabilize lesions, which may lower the atherosclerosis risk. Collectively, this diagram identifies Blimp-1-targeted pathways as novel therapeutic targets for the regulation of inflammation and oxidative stress in chronic inflammatory diseases. Solid lines indicate functions that have been confirmed in the cited literature, while dashed lines represent functions not directly verified by experiments in the cited literature but supported by evidence from other studies.

**Diabetes**: Blimp-1 may be a new target for the treatment of metabolic diseases. Blimp-1-targeted therapies are able to protect the antioxidant defenses by boosting SOD’s activity and mitigating the oxidative stress that compounds diabetic metabolic dysfunction [10]. Figure 2 shows the role of Blimp-1 across different immune cell types, illustrating its impact on ROS modulation and inflammation control, with therapeutic implications for metabolic diseases like diabetes and atherosclerosis.

**Cardiovascular diseases**: Blimp-1 is involved in influencing cardiovascular diseases, especially atherosclerosis, which is propelled by chronic inflammation and oxidative stress [10]. Strategies to increase Blimp-1’s activity in Th9 cells to block their pro-inflammatory and ROS-producing activity may enable the stabilization of atherosclerotic plaques and the risk of cardiovascular events. Lastly, Blimp-1 could also enhance SOD activity and reduce oxidative damage, thus improving endothelial function and preventing further cardiovascular complications.

**Figure 3:**
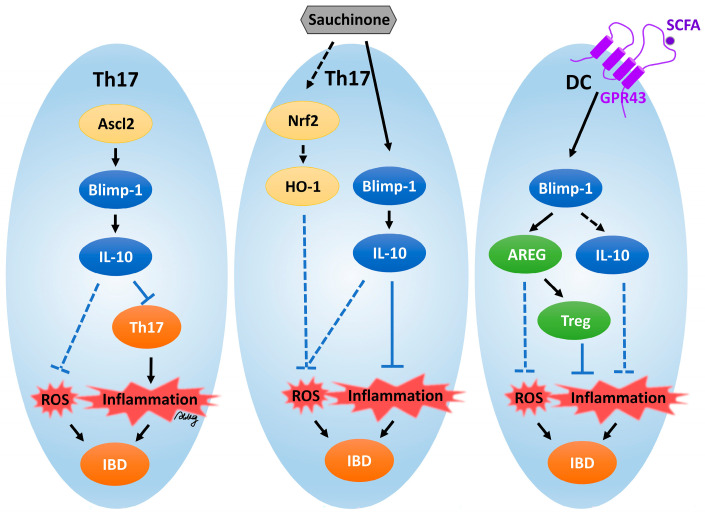
Blimp-1’s role in regulating oxidative stress and inflammation in immune cells associated with inflammatory bowel disease. This figure provides an overview of the regulatory pathways of Blimp-1 in Th17 cells and dendritic cells (DCs) relevant to inflammatory bowel disease (IBD). Blimp-1 induced by the Ascl2 pathway enhances IL-10 production in Th17 cells and suppresses inflammatory responses, leading to lessened IBD severity [9]. Sauchinone compounds activate Nrf2, producing HO-1, and additionally activating Blimp1 and generating IL-10 expression, mitigating IBD progress [8]. The activation of Blimp-1 by GPR43 stimulation with SCFAs in dendritic cells enhances AREG and IL-10 expression and supports Treg function [20]. The modulation of the immune response to ROS production and inflammation constitutes a potential therapeutic approach to mitigating IBD episodes. Solid lines indicate functions that have been confirmed in the cited literature, while dashed lines represent functions not directly verified by experiments in the cited literature but supported by evidence from other studies.

**Autoimmune diseases:** Considering their dual roles in autoimmune diseases like asthma and inflammatory bowel disease, such Blimp-1-targeted therapies may decrease inflammation and oxidative stress simultaneously. Blimp-1 may protect tissues against damage due to chronic inflammation by promoting the production of IL-10 and by enhancing the activity of antioxidant enzymes. For instance, increased Blimp-1 activity in Tregs and Th17 cells might significantly reduce joint inflammation and oxidative damage in inflammatory bowel disease and improve patient outcomes [8,9]. Figure 2 and Figure 3 illustrate the involvement of Blimp-1 in regulating oxidative stress and inflammation across various immune cell types, such as Th17, Tregs, and dendritic cells, highlighting its potential in treating autoimmune diseases like asthma and inflammatory bowel disease by enhancing IL-10 production and antioxidant activity.

The above animal studies have served as the basis for research on Blimp-1 in human pathologies to show its key role in immune regulation and its possible therapeutic effects in chronic diseases of inflammation. As a result, studies in chronic TCMR have shown that Blimp-1 represents a critical regulator in effector memory CD8^+^ T cells [140]. Its upregulation and co-localization with CD8^+^ T cells indicate that it participates in mechanisms for the maintenance of memory T cell activity and chronic kidney rejection pathogenesis. However, this suggests that Blimp-1 is a promising therapeutic target for the modulation of memory T cell responses in chronic TCMR. Likewise, Blimp-1 is an important regulator of Th9 cells and IL-9 production in DM-CHD [10]. In these patients, Blimp-1 expression is negatively associated with IL-9 levels; increased Th9 cell activity is associated with inflammation and oxidative stress. Therapeutically, enhancing Blimp-1’s expression or activity could be a strategy to suppress Th9-driven inflammation and slow disease progression. These findings are summarized in Table 2 and highlight the translational relevance of Blimp-1 as a therapeutic target for chronic diseases driven by immune and metabolic dysregulation.

### 6.3. Limitations and Future Directions

Despite providing a comprehensive review of Blimp-1’s regulatory role in antioxidant responses and metabolic reprogramming, several limitations in this review must be acknowledged. Due to unresolved questions and the narrow focus of this review, other potential roles of Blimp-1, such as in cancer and neurodegenerative diseases, remain underexplored. Secondly, the mechanistic pathway from Blimp-1 to metabolic and ROS regulation is still incompletely understood, and many reports are dependent on in vitro systems or animal models. There remains a challenge in the direct applicability of these studies to human pathologies. Thirdly, this review relied on the existing literature and therefore it may not reflect unpublished discoveries because the field develops at a fast pace. Finally, the therapeutic potential of Blimp-1 is emphasized, but practical applications and clinical trials of Blimp-1-modulating therapies are lacking, revealing the need for translational research. Potential future studies that could overcome these limitations should be explored. Precise gene editing and regulatory technologies should be developed to investigate the roles of Blimp-1 in a cell-type-specific manner in diverse immune and non-immune cell types. For example, this may include the identification of therapies that modulate Blimp-1’s function in specific immune cells to enhance antioxidant protection and reduce inflammation. The modulation of Blimp-1 in combination with existing antioxidant therapies could therefore be part of a synergistic approach to chronic diseases. Additionally, Blimp-1-targeting therapies might be combined with exogenous antioxidants or drugs that promote endogenous antioxidant enzyme activity, providing more complete protection against oxidative stress and the inflammatory response. Finally, small molecules, gene therapy, or biologics could also be explored to selectively upregulate Blimp-1 activity in disease-affected tissues in order to further optimize the therapeutic potential of the upregulation of Blimp-1. It is also necessary to validate Blimp-1’s pathways in human models using expanded experiments to confirm the results of preclinical studies. Moreover, researchers should test Blimp-1 modulation therapeutics and their combinational treatment with antioxidants using longitudinal studies and clinical trials, with the goal of establishing their efficacy and safety. Finally, the extended exploration of Blimp-1’s function in cancer and other neurodegenerative diseases is crucial to identify further therapeutic applications.

## 7. Conclusions

In this review, we have evaluated the role of transcription factor Blimp-1 in bridging metabolism and antioxidant pathways in chronic inflammatory diseases, including diabetes, cardiovascular disease, and autoimmune diseases. Blimp-1 is a key regulator of immune cell metabolism, inflammation, and oxidative stress, with potential as a therapeutic target for diseases exhibiting chronic inflammation and metabolic dysregulation. The key animal studies were summarized to ascertain the role of Blimp-1 in metabolism, antioxidant defense, and disease contexts in various immune cells (Table 3). They highlight Blimp-1’s ability to regulate IL-10 production in Tregs, Th17, and ILC2 cells, reducing pro-inflammatory pathways and promoting antioxidant enzyme expression, such as SOD and GPx. Furthermore, Blimp-1 regulates fatty acid oxidation and glycolysis to restrict immune cell metabolism, while avoiding lethal oxidative stress and preserving tissue homeostasis. Blimp-1’s activity might stabilize plaques in cardiovascular diseases such as atherosclerosis by reducing inflammation and ROS levels. Additionally, in autoimmune diseases, such as inflammatory bowel disease, the induction of Blimp-1 activity could limit inflammation and tissue damage. Studies in the future may involve searching for drugs to promote Blimp-1’s activity, together with currently used antioxidant therapies due to the possibility of synergistic advances. Blimp-1 may be another means to address the complicated intersection of inflammation, metabolic imbalance, and oxidative stress in the setting of chronic diseases, but further clinical trials and research are crucial.

## Figures and Tables

**Table 1:** Comparison of genes, protein structures, and functional features of human Blimp-1/*PRDM1* and mouse Blimp-1/*Prdm1.*

	Human Blimp-1/*PRDM1*	Mouse Blimp-1/*Prdm1*
Gene and Protein Naming	Gene: *PRDM1*; protein: PRDI-BF1 (commonly referred to as Blimp-1)	Gene: *Prdm1*; protein: Blimp-1
Gene Location	Chromosome 6: 105.99–106.11 Mb	Chromosome 10: 44.31–44.4 Mb
Gene Structure	7 exons; sequence and splicing may differ slightly	8 exons; overall structure is similar to human form but may have slight differences
Protein Structure	5 Krüppel-like zinc fingers; N-terminal and C-terminal acidic regions; proline-rich and PR domain; 67 fewer amino acids at N-terminal compared to mouse	5 Krüppel-like zinc fingers; N-terminal and C-terminal acidic regions; proline-rich and PR domain; 67 more amino acids at N-terminal compared to human
Functional Conservation	Highly conserved with 90% sequence similarity; represses Pax5 and Bcl6; interchangeable in experimental assays	
Differential Expression	Expressed in terminally differentiated immune cells such as plasma cells, effector T cells; antagonizes BCL6 in T cells	
Interaction with Co-Factors	Recruits chromatin-modifying co-factors (e.g., histone deacetylases, methyltransferases)	
Developmental Functions	Crucial for primordial germ cell formation, intestinal epithelial maturation, and immune system regulation; dose-dependent effects on development and immunity	

**Table 2:** Human studies investigating Blimp-1 regulation in chronic inflammatory diseases.

Disease	Human Sample	Method	Outcome	Reference
Chronic T-Cell-Mediated Rejection (TCMR)	Archival kidney biopsies (14 chronic, 10 acute)	Immunohistochemistry and real-time PCR (Blimp-1 mRNA)	Increased Blimp-1^+^/CD8^+^ T cells and upregulated OX40 signaling in chronic TCMR biopsies. Highlighted Blimp-1’s role in promoting effector memory T cell differentiation, contributing to the pathology of chronic TCMR.	Curci et al. [140]
Diabetic Coronary Heart Disease (DM-CHD)	Peripheral blood (18 DM-CHD, 18 non-DM-CHD, 12 healthy)	Real-time PCR (Blimp-1 mRNA) and flow cytometry (Th9 cells)	A negative correlation was observed between serum Blimp-1 mRNA levels and IL-9 levels, while flow cytometry analysis revealed an increased proportion of Th9 cells (IL-9^+^ CD4^+^ T cells) in DM-CHD patients compared to non-DM-CHD patients and healthy controls, implicating Blimp-1 in the regulation of IL-9 production in Th9 cells. The findings suggest that Blimp-1 acts as a regulatory factor that inhibits Th9 cell differentiation, potentially mitigating the pro-inflammatory and pro-atherogenic effects of Th9 cells in DM-CHD.	Chen et al. [10]

**Table 3:** Summary of Blimp-1’s roles in various immune cells involved in metabolism and antioxidant defense based on animal studies.

Immune Cell Type	Blimp-1 Function	Impact on Metabolic Pathway	Impact on Oxidative Stress	Disease Context	References
**ILC2 Cells**	Promotes IL-10 production in ILC2s	Shift from fatty acid oxidation to glycolysis	By regulating IL-10 production in ILC2s, Blimp-1 may indirectly impact oxidative stress	↓ Asthma	Howard et al. [21]
**Th9 Cells**	Inhibits Th9 differentiation	N/A	Blimp-1 reduces oxidative stress markers like MDA, enhances antioxidant enzymes like SOD	↓ Diabetic coronary heart disease	Chen et al. [10]
**Th17 Cells**	Promotes IL-10 production in Th17	N/A	1. By promoting IL-10 production in Th17 cells via Blimp-1 activation, Sauchinone may indirectly reduce oxidative stress 2. By promoting IL-10 production in Th17 cells via Blimp-1 activation, Ascl2 may indirectly reduce oxidative stress	↓ Inflammatory bowel disease	1. Xiao et al. [8] 2. Yi et al. [9]
**DCs**	Promotes antioxidant AREG expression via butyrate–GPR43 signaling in DCs	SCFA metabolism (butyrate)	AREG production promoted by Blimp-1 may potentially reduce oxidative balance	↓ Inflammatory bowel disease	Xiu et al. [20]
**aTregs**	Promotes IL-10 production in aTregs	Shift toward glucose oxidation occurs in absence of Blimp-1	By promoting IL-10 production in aTregs via Blimp-1 activation and suppressing adipocyte beiging and energy metabolism, Blimp-1 may indirectly influence oxidative stress	↑ Diet-induced obesity	Beppu et al. [5]
**TRM Cells**	Promotes TRM differentiation	N/A	Stabilizes plaques and may indirectly reduce macrophage-induced oxidative stress	↑ Plaque stability ↑ Protection in atherosclerosis	de Jong et al. [141]
**ASCs**	Promotes ASC differentiation	Upregulates OXPHOS	Supports ER stress response	N/A	Scharer et al. [142]

↓ indicates alleviation; ↑ indicates promotion; N/A indicates not available, meaning the data was either not collected, not recorded, or unavailable at the time of reporting.

## Abbreviations

Blimp-1: B-Lymphocyte-induced maturation protein 1; DCs: Dendritic cells; ILCs: Innate lymphoid cells; IL-10: Interleukin-10; Tregs: Regulatory T cells; Nrf2: Nuclear factor erythroid 2-related factor 2; ROS: Reactive oxygen species; RA: Rheumatoid arthritis; IBD: Inflammatory bowel disease; SOD: Superoxide dismutase; HO-1: Heme oxygenase-1; DM-CHD: Diabetic coronary heart disease; MDA: Malondialdehyde; AREG: Amphiregulin; ILC2s: Group 2 innate lymphoid cells; TNBS: Trinitrobenzene sulfonic acid; TNF-a: Tumor necrosis factor-a; HDACs: Histone deacetylases; IRF4: Interferon regulatory factor 4; STAT3: Signal transducer and activator of transcription 3; NF-κB: Nuclear factor kappa B; ZnNPs-H: Histidine-functionalized zinc oxide; MAPK: Mitogen-activated protein kinase; MMPs: Matrix metalloproteinases; mPTP: Mitochondrial permeability transition pores; LDL: Low-density lipoprotein; NO: Nitric oxide; GPx: Glutathione peroxidase; GM-LAB: Genetically modified lactic acid bacteria; SCFAs: Short-chain fatty acids; EGF: Epidermal growth factor; WAT: White adipose tissue; UCP1: Uncoupling protein 1; TRM: Tissue-resident memory; TCMR: T-cell-mediated rejection; FAO: Fatty acid oxidation; TCA: Tricarboxylic acid; ER: Endoplasmic reticulum; Xbp1: X-box binding protein 1; DIO: Diet-induced obesity; PPP: Pentose phosphate pathway; PKM2: Pyruvate kinase isozyme M2; NAMPT: Nicotinamide phosphoribosyltransferase; ASC: Antibody-secreting cells
